# Synthesis of Novel Triphenylamine-Based Organic Dyes with Dual Anchors for Efficient Dye-Sensitized Solar Cells

**DOI:** 10.1186/s11671-022-03711-6

**Published:** 2022-08-04

**Authors:** Samar E. Mahmoud, Ahmed A. Fadda, Ehab Abdel-Latif, Mohamed R. Elmorsy

**Affiliations:** Department of Chemistry, Faculty of Science, Mansoura University, El-Gomhoria Street, Mansoura, 35516 Egypt

**Keywords:** Di-cyanoacrylamide, Di-thiazolidine-5-one, Triphenylamine-based organic dyes, **N-719**

## Abstract

A new series of metal-free organic dyes (**SM1-5**) with dual anchors are synthesized for application in dye-sensitized solar cells (DSSC). Here, a simple triphenylamine (TPA) moiety serves as the electron donor, while di-cyanoacrylamide and di-thiazolidine-5-one units serve as the electron acceptors and anchoring groups. To understand the effect of dye structure on the photovoltaic characteristics of DSSCs, the photophysical and electrochemical properties, as well as molecular geometries calculated from density functional theory (DFT), are used for dyes **SM1-5**. The extinction coefficients of the organic dyes **SM1-5** are high (5.36–9.54 10^4^ M^−1^ cm^−1^), indicating a high aptitude for light harvesting. The photovoltaic studies indicated that using dye SM4 as a sensitizer showed a power conversion efficiency (PCE) of 6.09% (*J*_SC_ = 14.13 mA cm^−2^, *V*_OC_ = 0.624 V, FF = 68.89%). Interestingly, **SM4** showed the highest values of *V*_OC_ among all dyes, including **N-719**, due to its maximum dye coverage on the TiO_2_ surface, enhancing charge recombination resistance in the sensitized cell. The good agreement between the theoretically and experimentally obtained data indicates that the energy functional and basis set employed in this study can be successfully utilized to predict new photosensitizers' absorption spectra with great precision before synthesis. Also, these results show that bi-anchoring molecules have a lot of potentials to improve the overall performance of dye-sensitized solar cells.

**Supplementary Information:**

The online version contains supplementary material available at 10.1186/s11671-022-03711-6.

## Background

Since the forerunner work on dye-sensitized solar cells (DSSCs) by Grätzel in 1991, DSSCs have received much research attention due to their enormous advantages such as low cost, good performance in low-light environments, high discipline, environmental friendliness and colors customizable [1, 2]. The power conversion efficiency of DSCs increased from 7% in 1991 to 14.3% in 2015 using liquid electrolytes (LEs), TiO_2_ photoelectrode with nanoparticles sensitized with **ADEKA-1** and a carboxy-anchor organic dye, **LEG4** [3]. The details of the work of dye-sensitized solar cells were explained as reported [4, 5].

A DSSC consists of five major components: a semiconductor, a sensitizer, a counter electrode, a working electrode and an electrolyte [6, 7]. Semiconductors are ideal ingredients for DSSC anodes because of their large surface cross section area for photosensitizer anchoring. Photoanode materials include binary metal oxides such as TiO_2_, SnO_2_ and ZnO. In DSCs, TiO_2_ is the most distinguished candidate for the photoanode. TiO_2_ achieved high efficiencies due to its large band gap compared to traditional semiconductors and excellent physical properties such as chemical and optical stability and corrosion resistance [8, 9].

The counter electrode consists of a conducting layer on a plastic or glass substrate. Pt electrodes are commonly used due to their catalytic effect and high stability. Carbon black, silver and gold have also been tested as counter electrodes [10, 11].

The electrolyte is responsible for transporting charge between electrodes and continuously regenerates the dye during DSSC operation [12]. There are three types of electrolytes: liquid electrolytes, quasi-solid electrolytes and solid polymer electrolytes. The ${\mathrm{I}}_{3}^{ -}/{\mathrm{I}}^{-}$ redox couple was generally used in the electrolyte due to its high light absorption property and slow recombination reactions. Even though these liquid electrolytes lead to high efficiency, they cause problems such as leakage, being highly volatile, corrosion of metals and difficulties in the device sealing and fabrication processes. DSSCs based on a quasi-solid electrolyte can compete with liquid electrolytes in terms of PCE and exhibit better long-term stability. Solid-state conductors represent a strong solution to this shortage. Fenton and Wright proposed this type, which consists of complexes of alkali metal ions within a polymeric matrix. Park et al*.* added polar groups to the polymeric matrix to improve the ionic conductivity of solid polymer electrolytes [13–16].

The major patterns of photosensitizer are metal-free organic dyes [17]. Zeng et al. (2009) proposed that organic sensitizers containing triphenylamine units gave power conversion efficiencies of over 11% and 10% [18]. Yao et al. (2015) showed that the highest efficiency (*η*) of DSSCs employing a single metal-free organic dye has reached 13.0% and other dyes with PCE 11.8%. A rigidified phenanthrene-quinoxaline-based sensitizer was synthesized by Jiang et al. (2018), and the efficiency of its assembled DSSC reached 10.11% under AM1.5G irradiation [19, 20].

The chemical structure of the organic sensitizers plays an essential role in the features of photovoltaics in DSSCs [21]. One of the most important types of organic dyes is the conjugated donor–acceptor (D-π-A) because of its sturdy spectral response. The HOMO and LUMO levels of the sensitizers can be easily tuned by alternation of the donor, spacer, and acceptor moieties [22]. To increase the absorption of the metal-free organic dyes, many donor groups were introduced into structures such as triarylamine, carbazole, indole, coumarin and phenothiazine [23].

Triphenylamine units are more powerful electron donors than other substances, because of their fluorescence qualities and electron density. Furthermore, a well-known substance with a non-planar architecture that exhibits a stiff plane [24], three-dimensional steric, a hole transporting characteristic, light-harvesting features and decreased aggregation on semiconductor surfaces (TiO_2_) [25]. To improve DSSC photovoltaic efficiency, photosensitizers are required to have bathochromic shifts by introducing donating groups (alkoxy, alkyl) and aromatic groups to TPA that increase the HOMO energy orbital level [26] or introducing different anchoring groups (CN, CO and NH) that facilitate the electron injection from the donor moiety into the photoanode and decrease charge aggregation of the dye on TiO_2_ [27–32]. To improve the binding strength of dyes on TiO_2_, the incorporation of double electron-accepting groups into the organic donor structure to generate a double-anchored compound has been suggested, which exhibited higher device efficiency than single D-π-A dyes [33–39].

**Fig. 1:**
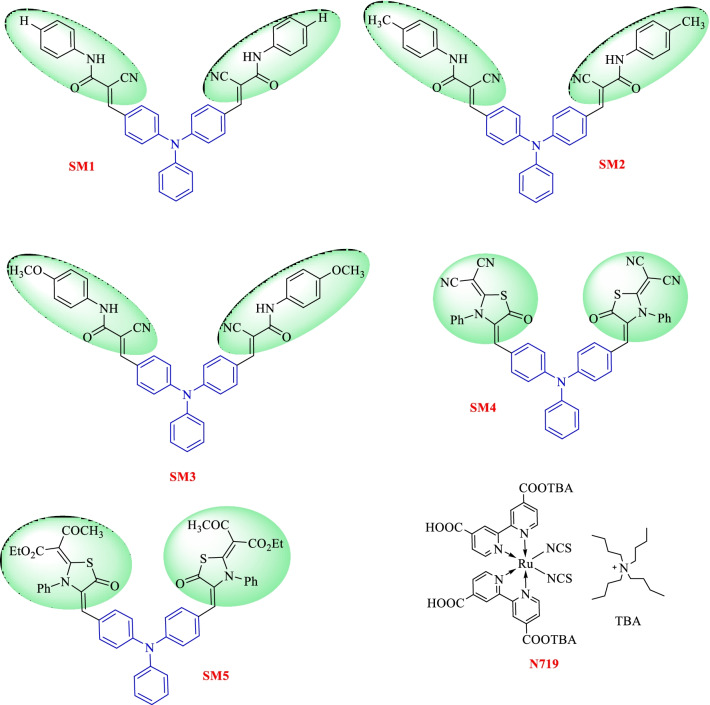
Molecular structures of the new sensitizers **SM1-5** and **N-719**

**Fig. 2:**
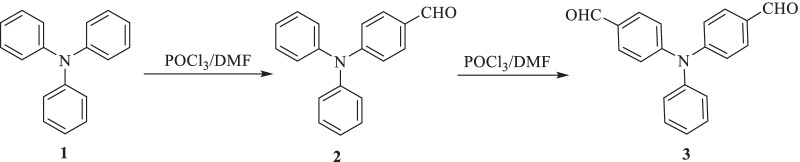
Synthesis of 4,4'-diformyl-triphenylamine (**3**)

**Fig. 3:**
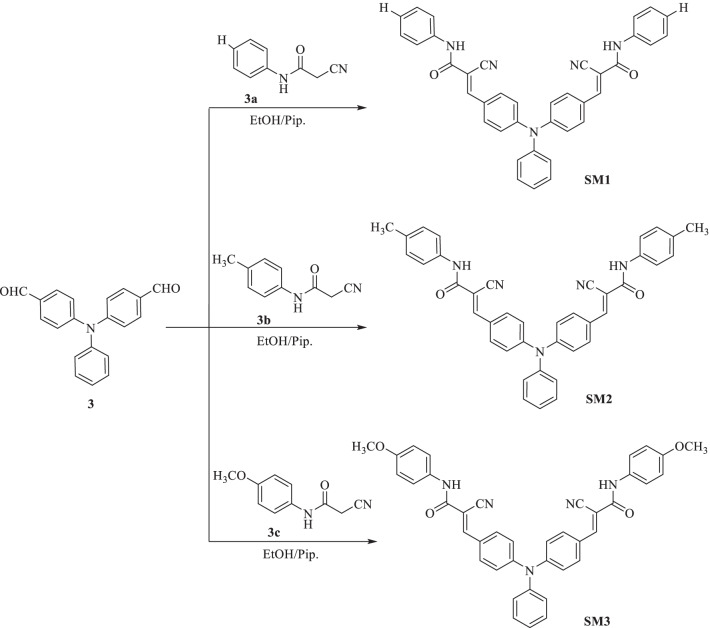
Synthesis of 3,3'-((phenylimino)bis(4,1-phenylene))bis(*N*-aryl-cyanoacrylamide) sensitizers **SM1-3**

**Fig. 4:**
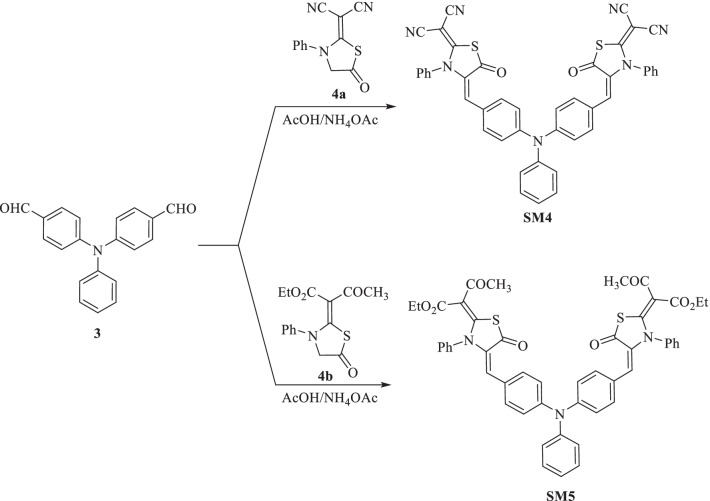
Synthesis of triphenylamine-thiazolidine-5-one sensitizers **SM4** and **SM5**

We launched five new di-anchoring compounds with a triphenylamine core as an electron donor, denoted as **SM1-5**. In this work, we used different and new di-anchoring structures, which were created with three-electron acceptors (cyanoacrylamide core, thiazolidine-5-one-dimolononitrile core, and thiazolidine-5-one-bis(3-oxobutanoate core)). Using organic photosensitizers with double electron acceptors/anchoring groups led to improved current efficiency as a result of increasing the molar extinction coefficient of the chromophore and also led to improved photovoltage because of the absorption maximum amount of sensitizer on the semiconductor surface. The power conversion efficiency in the DSSC is better than the single electron acceptor type [40, 41]. Figure 1 displays the molecular structures of the components **SM1-5, **which were developed and produced. Figures 2, 3 and 4 illustrate the synthetic paths. The structure of dyes **SM1-5** is confirmed by FTIR, ^1^H NMR, ^13^C NMR and MS. Their optical properties were calculated from UV–Vis absorption. The electronic distribution of HOMO/LUMO energy levels was studied using Gaussian 09. Compared to the standard dye N-719 [42], their photovoltaic performance and electrochemical impedance spectroscopy (EIS) were also studied.

## Experimental Section

### Materials and Instruments

The solvents and chemicals used in the synthesis of sensitizers **SM1-5** were purchased from Sigma-Aldrich. The melting points (degrees centigrade) were obtained on the Gallenkamp electric melting point device. ^1^H and ^13^C NMR spectra were measured in DMSO-*d*_6_ as a solvent at 500 MHz and 125 MHz, respectively, and obtained using JEOL’s NMR spectrometer. The Nicolet iS10 FTIR spectrometer was used to measure IR spectra (KBr disks). The UV–Visible spectra were measured by using a UV–Vis spectrophotometer (T80 series). Thermo Scientific GC/MS model ISQ was used to determine mass analyses. Elemental analysis was recorded by a PerkinElmer 2400 analyzer. The CV experiments were conducted by following the three-electrode system, consisting of platinum as counter, Ag/AgCl as a reference electrode, and glassy carbon was used as the working electrode. The data were recorded at a scan rate of 100 mV^−1^. Photocurrent–voltage characteristics of DSSCs were measured using a Keithley 2400 source meter under illumination of AM 1.5 G solar light from a solar simulator (SOL3A, Oriel) equipped with a 450 W xenon lamp (91160, Oriel). The incident light intensity was calibrated using a reference Si solar cell (Newport Oriel, 91150 V) to set 1 Sun (100 mW cm^−2^). The measurements were fully controlled under Oriel IV Test Station software. The electrochemical impedance spectra were measured with an impedance analyzer potentiostat (Bio-Logic SP-150) under illumination with a solar simulator (SOL3A, Oriel) equipped with a 450 W xenon lamp (91160, Oriel). EIS spectra were recorded over a frequency range of 100 mHz to 200 kHz at room temperature. The applied bias voltage was set at the *V*OC of the DSSCs with an AC amplitude of 10 mV. The electrical impedance spectra were fitted with Z-Fit software (Bio-Logic).

### Synthesis of Targeted Sensitizers SM1-5

#### Synthesis of 4-(Diphenylamino)benzaldehyde (2)

The title compound **2** was synthesized in good yield by formylation of triphenylamine (**1**) according to the reported Vilsmeier–Haack reaction [43].

#### Synthesis of 4,4'-Diformyl-triphenylamine (3)

In a 50-mL two-neck RB flask containing dry DMF (23 mL, 300 mmol), freshly purified POCl_3_ (24 mL, 260 mmol) was added dropwise at 0 °C, and the mixture was stirred in an argon atmosphere for 30 min at this temperature until the colored Vilsmeier salt completely precipitated. The solution of 4-(diphenylamino)benzaldehyde (**2**) (1 g, 4 mmol) dissolved in (10 mL) dry DMF was added, and the mixture of the reaction was stirred at 50 °C overnight. The mixture was cooled at room temperature, poured into ice water and neutralized to pH 7 with sodium acetate. The crude product was purified by column chromatography using the mixture of SiO_2_ and CH_2_Cl_2_ to produce yellow crystals.

90% yield; m.p. = 142–144 °C, lit. m.p. = 141–143 °C [43]. IR (ῡ, cm^−1^): 1691 (2 C=O). ^1^H NMR (*δ*/ppm): 7.16–7.19 (m, 7H, Ar–H), 7.39 (t, 2H, Ar–H), 7.77 (d, 4H, Ar–H), 9.8 (s, 2H, 2 CH=O). Analysis for C_20_H_15_NO_2_ (301.11): calculated: C, 79.72; H, 5.02; N, 4.65%, found: C, 79.55; H, 5.08; N, 4.74%.

### General Synthesis of 3,3'-((Phenylimino)bis(4,1-phenylene))bis(*N*-aryl-cyanoacrylamide) Sensitizers SM1-3

In a dry 50-mL RB flask, 4 mmol of each cyanoacetanilide derivative **3a–c** (namely 2-cyanoacetanilide (0.64 g, 4 mmol), 2-cyano-*p*-methylacetanilide (0.70 g, 4 mmol) and 2-cyano-*p*-methoxyacetanilide (0.76 g, 4 mmol)) was added to a solution of 4,4'-diformyl-triphenylamine (**3**) (0.60 g, 2 mmol) in 50 mL dry ethanol and 0.1 mL piperidine. The mixture was subjected to heating for 2 h. The crystalline finished product on hot was collected, rinsed with hot EtOH and refined by recrystallization to provide the sensitizers **SM1**, **SM2** and **SM3**, respectively.

#### 3'-((Phenylimino)bis(4,1-phenylene))bis(2-cyano-*N*-phenylacrylamide) (SM1)

Orange powder; 79% yield; m.p. = 265–266 °C. IR (ῡ, cm^−1^): 3332 (2 N–H), 2210 (2 C≡N), 1679 (2 C=O). ^1^H NMR (*δ*/ppm): 7.12 (t, 2H, Ar–H), 7.19 (d, 4H, Ar–H), 7.24 (d, 2H, Ar–H), 7.31–7.37 (m, 5H, Ar–H), 7.48 (t, 2H, Ar–H), 7.65 (d, 4H, Ar–H), 7.98 (d, 4H, Ar–H), 8.19 (s, 2H, 2 CH = C), 10.29 (s, 2H, 2 N–H). ^13^C NMR (*δ*/ppm): 104.09 (2C), 116.78 (2C), 120.63 (4C), 122.26, 122.59 (2C), 122.80, 124.30 (2C), 126.18 (2C), 126.48, 127.13 (2C), 128.77 (4C), 130.43 (2C), 131.38, 132.21 (2C), 138.35 (2C), 144.90, 149.64 (2C), 149.83 (2C), 151.36, 160.86 (2C). Mass analysis (*m*/*z*, %): 585 (M^+^, 24.27), 427 (42.52), 346 (58.22), 295 (30.47), 265 (27.16), 157 (54.05), 117 (37.70), 92 (83.55), 79 (27.02). Analysis for C_38_H_27_N_5_O_2_ (585.22), calculated: C, 77.93; H, 4.65; N, 11.96%, found: C, 77.79; H, 4.70; N, 11.87%.

#### 3'-((Phenylimino)bis(4,1-phenylene))bis(2-cyano-*N*-(p-tolyl)acrylamide) (SM2)

Orange powder; 76% yield; m.p. above 300 °C. IR (ῡ, cm^−1^): 3380 (2 N–H), 2208 (2 C≡N), 1689 (2 C=O). ^1^H NMR (*δ*/ppm): 2.27 (s, 6H, 2 CH_3_) 7.14–7.25 (m, 10H, Ar–H), 7.32 (t, 1H, Ar–H), 7.46 (q, 2H, Ar–H), 7.53 (d, 4H, Ar–H), 7.96 (d, 4H, Ar–H), 8.17 (s, 2H, 2 CH = C), 10.19 (s, 2H, 2 N–H). ^13^C NMR (*δ*/ppm): 21.03 (2C), 105.10 (2C), 114.08 (4C), 116.88 (2C), 121.98 (2C), 122.38 (4C), 122.98 (2C), 123.17, 125.24 (2C), 126.34, 127.15 (2C), 130.11 (2C), 131.05 (2C), 132.37 (2C), 145.95 (2C), 149.76 (2C), 149.98 (2C), 155.94 (2C), 161.03 (2C). Mass analysis (*m*/*z*, %): 613 (M^+^, 10.42), 581 (18.24), 403 (21.88), 303 (34.30), 286 (44.03), 282 (99.22), 188 (40.58), 82 (58.71), 60 (62.61), 41 (67.21), 40 (100.00). Analysis for C_40_H_31_N_5_O_2_ (613.25): calculated: C, 78.28; H, 5.09; N, 11.41%, found: C, 78.38; H, 5.05; N, 11.33%.

#### 3'-((Phenylimino)bis(4,1-phenylene))bis(2-cyano-*N*-(4-methoxyphenyl)acrylamide) (SM3)

Dark orange powder; 74% yield; m.p. = 274–276 °C. IR (ῡ, cm^−1^): 3360 (2 N–H), 2206 (2 C≡N), 1680 (2 C=O). ^1^H NMR (*δ*/ppm): 3.73 (s, 6H, 2 OCH_3_), 6.92 (d, 4H, Ar–H), 7.18 (d, 4H, Ar–H), 7.23 (d, 2H, Ar–H), 7.31 (t, 1H, Ar–H), 7.48 (t, 2H, Ar–H), 7.55 (d, 4H, Ar–H), 7.96 (d, 4H, Ar–H), 8.16 (s, 2H, 2 CH = C), 10.15 (s, 2H, 2 N–H). ^13^C NMR (*δ*/ppm): 55.21 (2C), 104.10 (2C), 113.86 (4C), 116.84 (2C), 122.18, 122.33 (4C), 122.58 (3C), 122.87, 126.24 (2C), 126.44, 127.10 (2C), 130.41 (2C), 131.31 (2C), 132.15 (3C), 144.93, 149.55 (2C), 149.58 (2C), 155.97 (2C), 160.42 (2C). Mass analysis (*m*/*z*, %): 645 (M^+^, 41.66), 566 (46.65), 529 (58.87), 518 (63.83), 508 (73.91), 458 (100.00), 381 (61.55), 347 (48.00), 298 (86.32), 250 (59.37), 237 (48.43), 188 (83.61), 178 (95.65), 160 (90.38), 131 (62.40), 118 (87.81), 102 (49.64), 67 (41.52). Analysis for C_40_H_31_N_5_O_4_ (645.24): calculated: C, 74.40; H, 4.84; N, 10.85%, found: C, 74.58; H, 4.76; N, 10.97%.

### General Synthesis of Triphenylamine-thiazolidine-5-one Sensitizers SM4 and SM5

In a dry 50-mL RB flask, 4 mmol of each thiazolidine-5-one derivative **4a** or **4b** [namely, 2-(5-oxo-3-phenylthiazolidine-2-ylidene) malononitrile (0.96 g, 4 mmol), ethyl-3-oxo-2-(5-oxo-3-phenylthiazolidine-2-ylidene)butaneperoxoate (1.22 g, 4 mmol)] was added to a mixture of 4,4'-diformyl-triphenylamine (**3**) (0.60 g, 2 mmol) and ammonium acetate (0.39 g, 5 mmol) in 30 ml of glacial acetic acid. The mixture was subjected to reflux for 2 h. The collected precipitate was purified and dried to **SM4** and **SM5**, respectively.

#### 2'-((((Phenylimino)bis(4,1-phenylene))bis(methanylylidene))bis(5-oxo-3phenylthiazolidine-4,2-diylidene))dimalononitrile (SM4)

Red powder; 85% yield; m.p. = 259–260 °C. IR (ῡ, cm^−1^): 2215 (2 C≡N), 1721 (2 C=O). ^1^H NMR (*δ*/ppm): 7.20 -7.26 (m, 6H, Ar–H), 7.32 (q, 1H, Ar–H), 7.48 (q, 2H, Ar–H), 7.56 -7.60 (m, 10H, Ar–H), 7.71 (d, 4H, Ar–H), 8.00 (s, 2H, 2 CH = C). ^13^C NMR (*δ*/ppm): 110.00, 114.05, 115.03 (2C), 122.13, 122.96 (3C), 123.31, 126.72 (2C), 127.17 (2C), 127.23 (2C), 129.31 (4C), 129.60 (4C), 130.45 (2C), 131.29, 131.37, 132.55 (3C), 133.27 (2C), 134.93 (2C), 144.99, 148.62 (2C), 162.34, 165.97 (2C), 167.13 (2C), 191.22, 191.46. Mass analysis (*m*/*z*, %): 747 (M^+^, 14.99), 710 (34.96), 675 (40.09), 594 (29.54), 461 (50.93), 356 (65.05), 347 (40.19), 225 (27.23), 175 (34.60), 64 (54.51), 62 (57.61), 51 (62.00), 44 (100.00), 43 (97.07). Analysis for C_44_H_25_N_7_O_2_S_2_ (747.15): calculated: C, 70.67; H, 3.37; N, 13.11%, found: C, 70.98; H, 3.45; N, 13.26%.

#### Diethyl 2,2'-((((phenylimino)bis(4,1-phenylene))bis(methanylylidene))bis(5-oxo-3-phenylthiazolidine-4,2-diylidene))-bis(3-oxobutanoate) (SM5)

Red powder; 87% yield; m.p. = 255–256 °C. IR (ῡ, cm^−1^): 1709 (2 C=O), 1644 (2 C=O). ^1^H NMR (*δ*/ppm): 1.03 (t, *J* = 7.50 Hz, 6H, 2 CH_3_), 2.08 (s, 6H, 2 COCH_3_), 4.27 (q, 4H, 2 CH_2_), 7.21 (d, 6H, Ar–H), 7.28 (t, 1H, Ar–H), 7.34 (d, 4H, Ar–H), 7.44–7.52 (m, 8H, Ar–H), 7.69 (d, 4H, Ar–H), 7.73 (s, 2H, 2 CH=C). ^13^C NMR (*δ*/ppm): 14.00 (2C), 29.90 (2C), 62.09 (2C), 110.14, 114.96 (2C), 116.26 (2C), 122.21, 122.97 (4C), 123.56, 125.93 (2C), 126.58 (2C), 127.31 (2C), 129.29 (4C), 130.10 (4C), 131.55, 132.48 (2C), 133.51 (2C), 135.00 (2C), 146.05, 148.26 (2C), 149.35, 152.16, 162.83, 166.15 (2C), 167.98 (2C), 192.30 (2C). Mass analysis (*m*/*z*, %): 876 (M^+^, 30.24), 849 (39.53), 777 (30.21), 655 (26.12), 640 (26.12), 507 (26.51), 476 (29.77), 376 (31.21), 372 (60.01), 269 (47.79), 216 (27.95), 151 (26.25), 148 (40.27), 91 (60.27). Analysis for C_50_H_41_N_3_O_8_S_2_ (875.23): calculated: C, 68.56; H, 4.72; N, 4.80%, found: C, 68.79; H, 4.62; N, 4.91%.

## Results and Discussion

### Synthesis and Structure Characterization of Sensitizers SM1-5

The synthetic routes for the 3,3'-((phenylimino) bis(4,1-phenylene)) bis(*N*-aryl-cyanoacrylamide) sensitizers **SM1-3** are depicted in Figs. 2 and 3. Ren et al. were able to monoformylate triphenylamine (**1**) to make 4-(diphenylamino)benzaldehyde (**2**) [43]. According to the reported procedure [44], 4-(diphenylamino)benzaldehyde (**2**) then goes through another formylation to prepare 4,4'-diformyl-triphenylamine (**3**).

Finally, Knoevenagel condensation reaction of 4,4'-diformyl-triphenylamine (**3**) with cyanoacetanilide derivatives **3a-c** in dry ethanol and drops of piperidine as a catalyst furnished the targeted 3,3'-((phenylimino)bis(4,1-phenylene))bis(*N*-aryl-cyanoacrylamide) sensitizers **SM1-3** with yields of 79%, 76% and 74%. The cyanoacetylation of aniline, *p*-toluidine and *p*-anisidine with 1-cyanoacetyl-3,5-dimethylpyrazole in boiling dioxane was used to make the cyanoacetanilide derivatives **3a–c** [45].

The structures of these sensitizers are confirmed by elemental and spectroscopic analyses (IR, ^1^H NMR, ^13^C NMR and MS). The chemical structure of dye **SM1** was confirmed by characteristic absorption bands from the IR spectrum: N–H groups at 3332 cm^−1^; cyano groups (C≡N) at 2210 cm^−1^; and a broad absorption band for the carbonyl groups (C=O) at 1679 cm^−1^. Its ^1^H NMR spectrum showed a singlet for two olefinic protons at *δ* 8.19 ppm and a singlet at *δ* 10.29 ppm for two protons of two N–H groups. The aromatic protons resonate as doublet, triplet and multiplet signals in the region from *δ* 7.12 to 7.98 ppm. Its ^13^C NMR spectrum exhibited the characteristic carbon signals at *δ* 116.78 ppm for (2C≡N), *δ* 149.83 ppm for (2C=C) and *δ* 160.86 ppm for (2C=O).

Also, the IR spectrum of **SM2** exhibited absorption bands at 3380 cm^−1^, 2208 cm^−1^ and 1689 cm^−1^ for the N–H, C≡N and C=O functional groups. Its ^1^H NMR spectrum showed singlet for six protons at 2.27 ppm is distinct for two methyl groups, a singlet for two olefinic protons and two protons of N–H groups at *δ* 8.17 and 10.19 ppm, respectively. Its mass spectrum displayed a molecular ion peak at *m*/*z* = 613, corresponding to the general formula C_40_H_31_N_5_O_2_. The structure of **SM3** was proved from the IR spectrum that showed three characteristic absorption bands at 3360 cm^−1^, 2206 cm^−1^ and 1680 cm^−1^ for N–H, C≡N and C=O groups, respectively. The ^1^H NMR spectrum showed a singlet at *δ* 3.73 ppm for six protons that is assignable to methoxy groups, singlet for two olefinic protons at *δ* 8.16 ppm and two protons of N–H groups at *δ* 10.15 ppm. Its ^13^C NMR spectrum showed signals for two similar carbons of methoxy, nitrile and carbonyl groups at *δ* 55.21, 116.84 and 160.42 ppm, respectively. It had a molecular ion peak at *m*/*z* = 645, which corresponded to a molecular formula of C_40_H_31_N_5_O_4_.

The synthetic pathway of triphenylamine-thiazolidine-5-one dyes **SM4** and **SM5** is displayed in Fig. 4 according to the Knoevenagel condensation reaction between each thiazolidine-5-one derivative **4a** or **4b** [46] and 4,4'-diformyl-triphenylamine (**3**). The reaction proceeds by heating in acetic acid containing ammonium acetate to furnish the targeted dyes **SM4** and **SM5** in high yields of 85% and 87%, respectively. The chemical structure of organic synthesizers was identified by performing various spectra data. The IR spectrum of dye **SM4** exhibited a broad absorption at 2215 cm^−1^ and 1721 cm^−1^ for (C≡N) and (C=O) groups. Its ^1^H NMR spectrum showed a singlet at *δ* 8.00 ppm for the olefinic protons. Its ^13^C NMR spectrum showed signals of two analogical carbons of (C≡N) at *δ* 115.03 ppm and two signals of (C=O) at *δ* 191.22 and 191.46 ppm, respectively. Its mass spectrum exhibited a molecular ion peak at *m*/*z* = 747, which corresponds to the molecular formula C_44_H_25_N_7_O_2_S_2_. Further, the IR spectrum of **SM5** displayed the characteristic absorption of two carbonyl groups at 1709 and 1644 cm^−1^. Its ^1^H NMR spectrum showed two singlet signals of methyl and olefinic protons at *δ* 2.08 and 7.73 ppm, a triplet at *δ* 1.03 for six protons (two methyl groups) and a quartet for four protons at *δ* 4.27 ppm (two methylene groups). Its mass spectrum showed that the molecular ion peak was at *m*/*z* = 876, which is the same as C_50_H_41_N_3_O_8_S_2_ as a molecular formula.

### Optical Behavior and Electrochemical Properties of Dyes

**Fig. 5:**
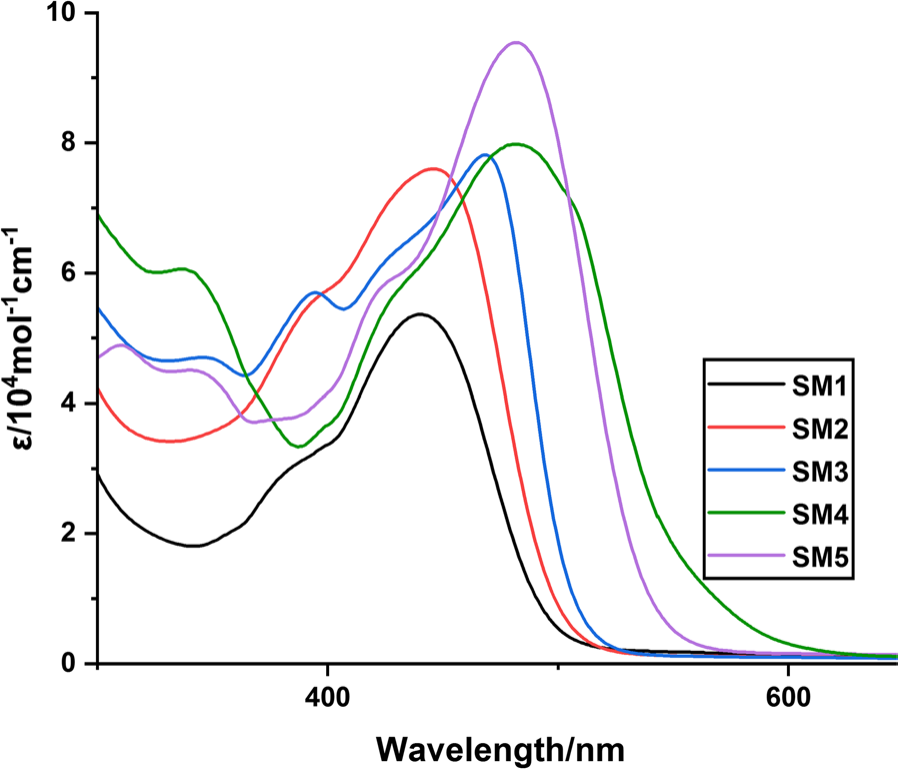
UV–Vis absorption spectra of **SM1-5**measured in DMF (2 × 10^–5^ M)

The UV–Vis absorption spectra of five new organic sensitizers **SM1-5** in dimethylformamide (DMF) solution with a concentration (2 × 10^–5^ M) are recorded in Fig. 5. Table 1 contains typical spectral data. All dyes possess different absorption bands; the region with a lower wavelength (300–430 nm) is assigned to the π–π* transitions, and another region with a longer wavelength (440–560 nm) is due to intramolecular (ICT) from an arylamine donating moiety (triphenylamine) to an electron acceptor (cyanoacrylamide moiety and thiazolidine-5-one derivatives). The maximum absorption wavelengths of five dyes **SM1-5** are 441 nm (molar extinction coefficient/ε = 5.36 × 10^4^ M^−1^ cm^−1^), 446 nm (molar extinction coefficient/ε = 7.60 × 10^4^ M^−1^ cm^−1^), 470 nm (molar extinction coefficient/ε = 7.75 × 10^4^ M^−1^ cm^−1^) and 483 nm (molar extinction coefficient/ε = 7.98 × 10^4^ M^−1^ cm^−1^), 483 nm (molar extinction coefficient/ε = 9.54 × 10^4^ M^−1^ cm^−1^). Further, the onset of the highest absorption band wavelength (*λ*_onset_) of the UV–visible spectra can also be used to compute *E*_0-0_ (energy gap) [47]. *E*_0-0_ values for compounds **SM1-5** are 2.50, 2.44, 2.41, 2.24 and 2.28 eV, respectively.

**Table 1:** Absorption data for sensitizers **SM1-5**

Sensitizer	Absorption *λ*_max_ (nm)	*ε* (10^4^ M^−1^ cm^−1^)	*λ*_onset_ (nm)	Practical *E*_0-0_ (eV)
**SM1**	375, 441	2.69, 5.36	494	2.50
**SM2**	389, 446	5.58, 7.60	508	2.44
**SM3**	393, 470	5.68, 7.75	514	2.41
**SM4**	424, 483	5.73, 7.98	553	2.24
**SM5**	419, 483	5.57, 9.54	543	2.28

From Table 1, **SM4** and **SM5**, which have the thiazolidine-5-one moiety, exhibited bathochromic shift and lower energy compared to cyanoacrylamide units. This bathochromic shift provides good indication for gathering photons from the solar light, which results in better photovoltaic performance. Furthermore, the molar extinction coefficients of the absorption maximum wavelength are substantially bigger than for the other components **SM1-3**, indicating a strong aptitude for light harvesting. The lowest energy gap of **SM4** is attributed to the lower resonance energy, high conjugation system and presence of an electron-withdrawing group (CN) attached to the thiazolidine moiety as an anchor, which facilitates the charge transfer. On the other hand, **SM3**, which has cyanoacrylamide, appeared to have a bathochromic shift compared to cyanoacrylamides (**SM1** and **SM2**) due to the strong electron-donating group (methoxy group). The lack of a substituent on the phenyl group of the cyanoacrylmide is what makes **SM**1 bad at absorbing things.

**Fig. 6:**
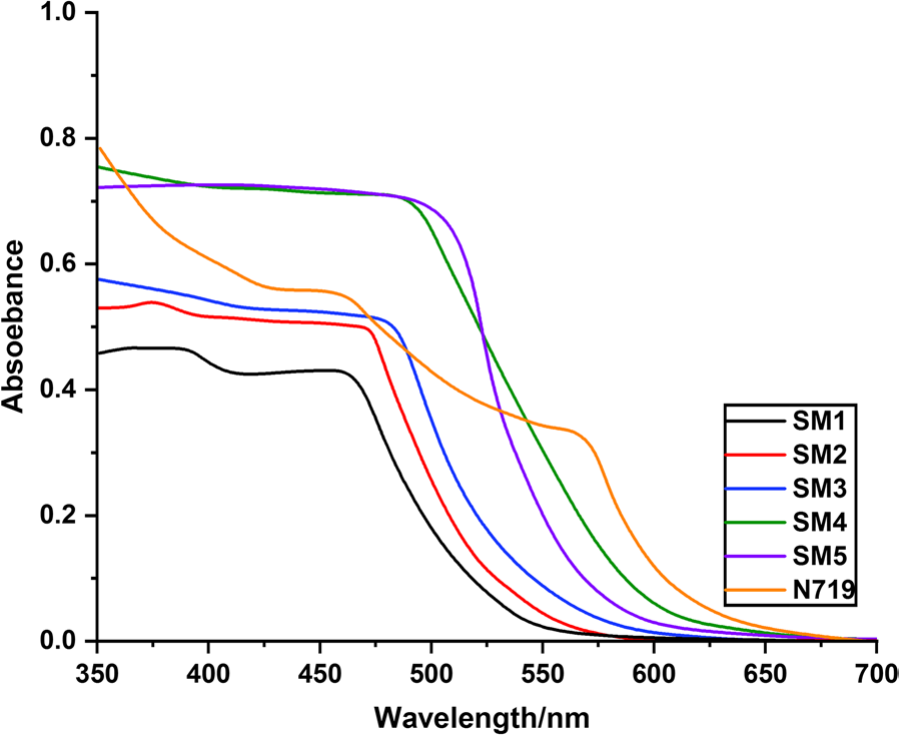
UV–Vis spectra of **SM1-5** and **N-719** adsorbed on TiO_2_ films

The normalized spectral data for the five components of TiO_2_ particles are shown in Fig. 6. The absorption bands of the five structures on TiO_2_ particle surfaces were redshifted relative to the spectra in the DMF solution. That may be related to the significant interactions among different components and the TiO_2_ particles, particularly the creation of *J*-type aggregation. Additionally, as compared to **SM2-5** dye, **SM1** dye has a higher, redshifted merit, suggesting that **SM1** has a higher ability to cluster on TiO_2_. Interestingly, the absorption spectra of **SM4** on the TiO_2_ surface are identical with those of the dye in solution, demonstrating that the dye does not aggregate.

Cyclic voltammetry (CV) measurements were performed in DMF solution to estimate the thermodynamic probability of electron injection and dye regeneration. Ferrocene (0.4 V vs. normal hydrogen electrode (NHE)) is served as an external reference to regulate the redox potential. Additional file 1: Fig. 23 is the cyclic voltammetry curves of **SM1-5**. The corresponding data are displayed in Table 2. The ground state oxidation potential (GSOP) levels for **SM1-5** were generally considered acceptable and higher than the iodine/triiodide redox overall value (− 5.2 eV), which confirmed that there is enough thermodynamic driving force to regenerate the dye by replenishing the hole through electron donation from the ${\mathrm{I}}_{3}^{ -}/{\mathrm{I}}^{-}$ redox couple. Moreover, the estimated excited state potentials (ESOP) for **SM1-5**, computed from GSOP ^_^ *E*_0-0_, are − 3.36 eV, − 3.31 eV, − 3.27 eV, -− 3.16 eV and − 3.21 eV, respectively.

**Table 2:** Electrochemical data for dyes **SM1-5**

Dye	Practical parameters (eV)			Calculated parameters (eV)		
	*E*_0-0_	HOMO	LUMO	*E*_0-0_	HOMO	LUMO
**SM1**	2.50	− 5.86	− 3.36	2.64	− 5.89	− 3.25
**SM2**	2.44	− 5.75	− 3.31	2.69	− 5.87	− 3.18
**SM3**	2.41	− 5.68	− 3.27	2.58	− 5.73	− 3.15
**SM4**	2.24	− 5.40	− 3.16	2.36	− 5.35	− 2.99
**SM5**	2.28	− 5.49	− 3.21	2.39	− 5.43	− 3.04

**Fig. 7:**
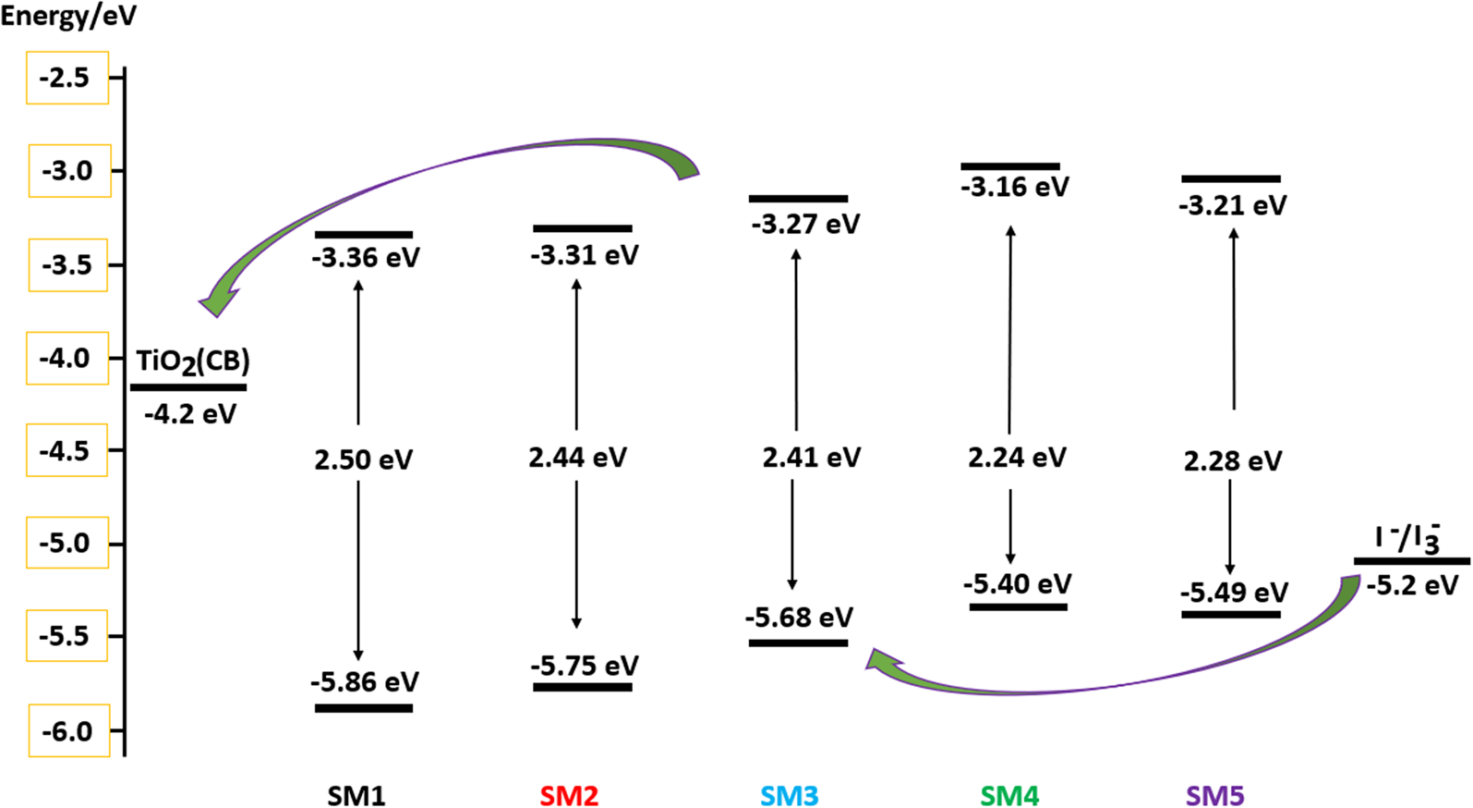
Energy level diagram of dyes **SM1-5**

The ESOP values of structures **SM1-5** are clearly above the conduction band (CB) of TiO_2_ (− 4.2 eV). This means that it is thermodynamically spontaneous for an electron to move from the excited state of the dyes into the CB of TiO_2_. Furthermore, **SM4** has a more negative free energy of electron injection than other dyes, which indicates that the light-excited electrons are injected more efficiently in the case of **SM4**. These results indicate that these compounds have the prerequisites to be employed as dye-sensitized solar cells. The energy level diagram of dyes **SM1-5** is shown in Fig. 7.

### Theoretical Investigation

**Fig. 8:**
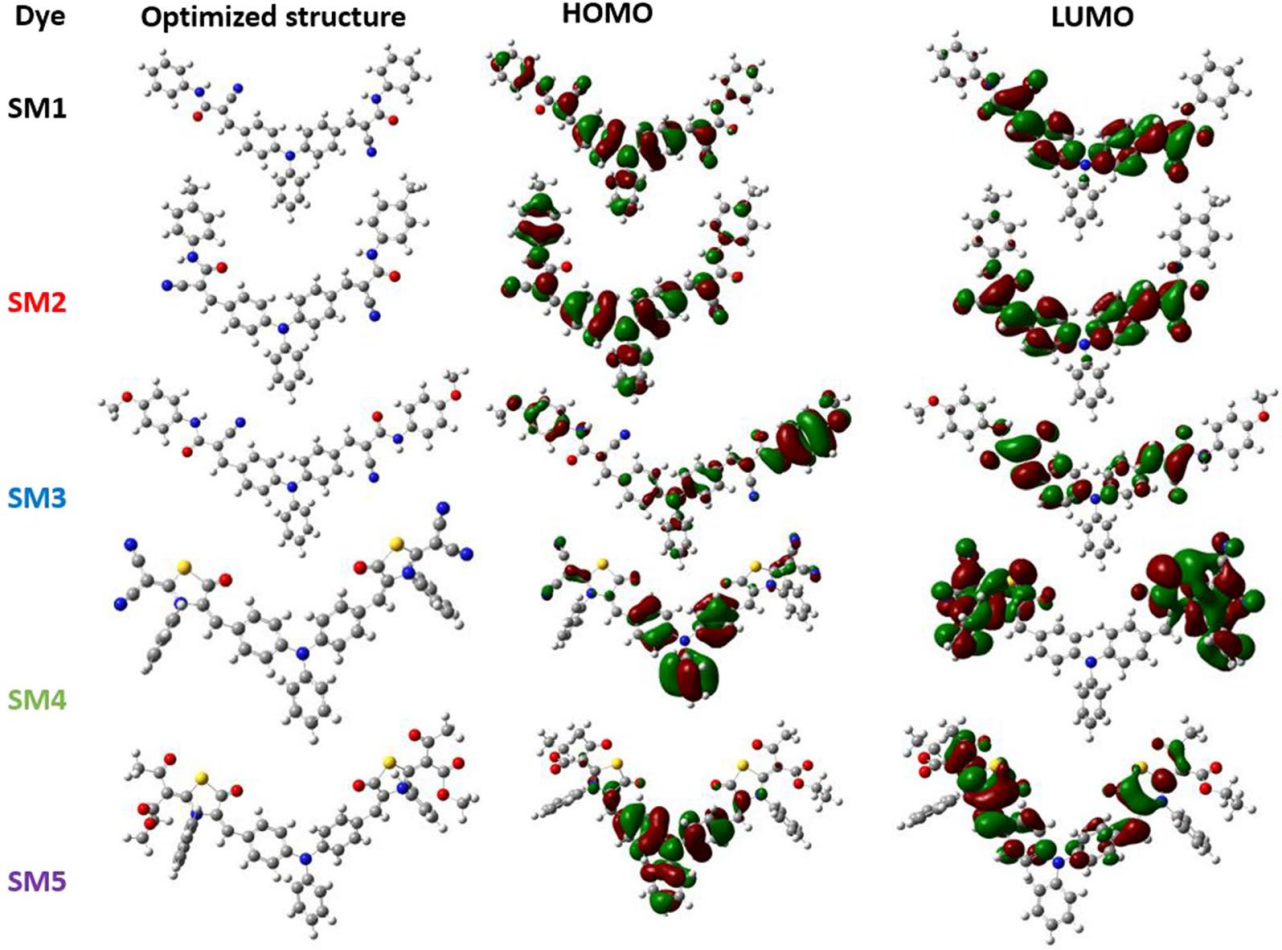
Optimized structures and molecular orbital distributions of SM1-5

The metal-free dyes **SM1-5** were optimized using DFT simulations with the Gaussian 09 software [48, 49] to explore their molecular geometry and electron circulation. The computations were carried out using the B3LYP exchange correlation functional using the basis set 6-311G (d, P) [50, 51]. Figure 8 depicts the optimized structures and molecular orbital distributions of **SM1-5**. As shown in Fig. 8, the electron distribution of the highest occupied molecular orbital (HOMO) for **SM1-5** may be detected across the molecules, notably in conjugated systems, while at the lowest unoccupied molecular orbital (LUMO), the distribution of the electrons for the five dyes is located over the acceptor moieties, especially on the cyanoacrylamides to **SM1-3** and over the thiazolidine-5-one moiety to **SM4** and **SM5**. This leads to a greater electronic coupling between the excited electrons of the dye in the LUMO and the unfilled *d*-orbitals of the semiconductor.

### Photovoltaic Performances of DSSCs

**Fig. 9:**
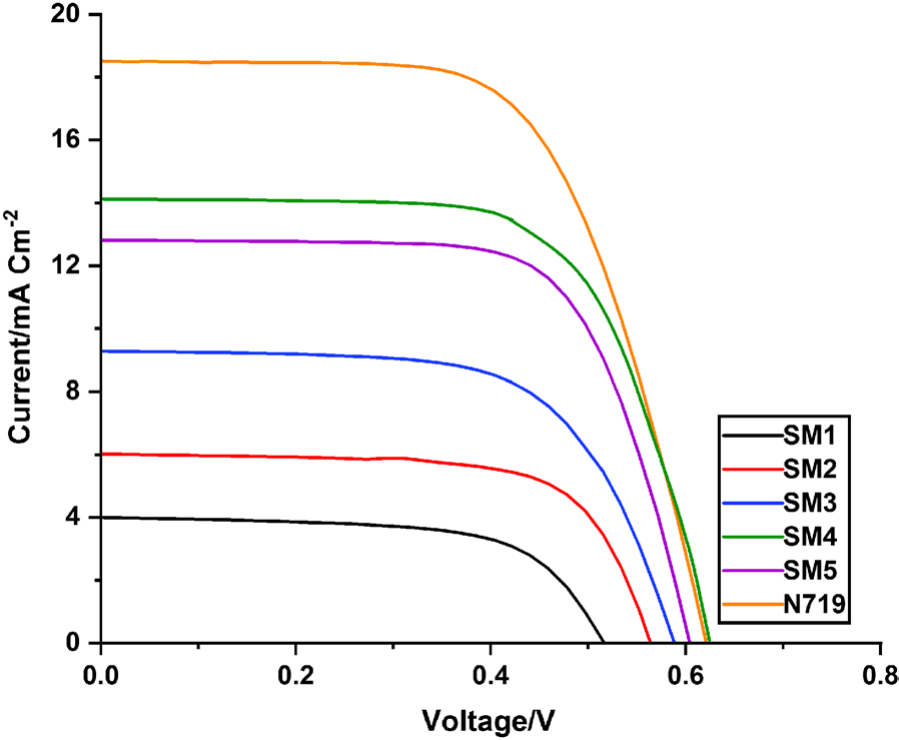
*J–V* characteristics curves of the DSSCs fabricated using **SM1-5** and **N719**

Under the illumination of AM 1.5G solar light from a solar simulator (SOL3A, Oriel), the photovoltaic results of DSSCs designed and manufactured utilizing **SM1-5** and the benchmark dye **N-719** on the TiO_2_ anode material using 10 mM chenodeoxycholic acid (CDCA) as a co-adsorbent were investigated using a Keithley 2400 source meter. The DSSC devices were fabricated according to the technique outlined in the Additional file 1 [52–54] with the goal of confirming the structure–performance assembly for compounds **SM1-5**. Furthermore, the co-adsorbent CDCA works as a proton buffer for the sensitizers, regulating dye proton concentration and so permitting enhanced dye adsorption on the anode nanoparticles [55, 56]. It also aids in the suppression of dye accumulation and covering of the TiO_2_ surface, resulting in a reduction in the redox electrolyte's electron recombination process. The presence of new acceptor segments in the chemical modification of **SM1-5** has had a significant impact on photovoltaic parameters like open-circuit photovoltage (*V*_OC_), short-circuit photocurrent density (*J*_SC_), fill factor (FF) and overall solar light to electricity conversion efficiency (*η*) of the sensitized cells in the proposed investigation. The current–voltage (*J–V*) characteristics curves of the DSSCs made with **SM1-5** are shown in Fig. 9, and the results are summarized in Table 3.

**Table 3:** *J–V* parameters of **SM1-5** and **N-719**

Dye	*V*_OC_^a^ (*V*_OC_^b^) (V)	*J*_SC_^a^ (*J*_SC_^b^) (mA cm^−2^)	FF^a^ (FF^b^) (%)	*η*^a^ (*η*^b^) (%)	Dye loading (mol cm^−2^)
**SM1**	**0.515** (0.498 ± 0.017)	**4.06** (3.94 ± 0.12)	**63.81** (63.5 ± 0.31)	**1.33** (1.24 ± 0.09)	**2.19** × 10^–8^
**SM2**	**0.563** (0.538 ± 0.022)	**6.06** (5.84 ± 0.22)	**68.53** (68.35 ± 0.18)	**2.34** (2.15 ± 0.19)	**4.15** × 10^–8^
**SM3**	**0.587** (0.572 ± 0.015)	**9.3** (9.17 ± 0.13)	**64.37** (64.06 ± 0.31)	**3.52** (3.36 ± 0.16)	**5.14** × 10^–8^
**SM4**	**0.624** (0.614 ± 0.009)	**14.13** (14.06 ± 0.06)	**68.89** (68.55 ± 0.33)	**6.09** (5.91 ± 0.18)	**4.21** × 10^–7^
**SM5**	**0.602** (0.581 ± 0.018)	**12.83** (12.54 ± 0.29)	**68.95** (68.55 ± 0.39)	**5.34** (4.89 ± 0.44)	**3.87** × 10^–7^
**N719**	**0.620** (0.605 ± 0.049)	**18.51** (18.34 ± 0.38)	**63.47** (63.21 ± 0.26)	**7.3** (7.01 ± 0.28)	**1.65** × 10^–6^

*V*_*OC*_: the open circuit voltage, *J*_*SC*_: the short current density, *FF*: the fill factor, and η: the efficiency

^a^The best device parameters (listed in the manuscript)

^b^The average device parameters (obtained from four devices)

The photovoltaic efficiency of the fabricated cells was found to be as follows: **SM4** > **SM5** > **SM3** > **SM2** > **SM1**. It is also important to mention that the thiazolidine-5-one **SM4** and **SM5** showcased higher performance than the cyanoacrylamides **SM1-3**. The highest performance was achieved using **SM4** as follows: *η* = (6.09%), superior *J*_SC_ (14.13 mA cm^−2^), and the greatest photovoltage value (0.624 V). The **SM1**, **SM2**, **SM3** and **SM5** fabricated devices showed *η* values of 1.33%, 2.34%, 3.52% and 5.34%, respectively. The higher performance of thiazildine-5-one dyes (**SM4**, **5**) compared to cyanoacrylamide dyes (**SM1-3**) is attributed to the maximum values of the *J*_SC_ and *V*_OC_. The remarkably enhanced *J*_SC_ related to their higher dye loading than the cyanoacetamide-based dyes **SM1-3**. Generally, dye loading studies are performed in the quest to understand the difference in efficiency with the variation in the anchoring groups. Keeping this in view, to estimate the total amount of dye adsorbed on the TiO_2_ surface, desorption of the dye from the TiO_2_ was done using 0.1 M NaOH in DMF/H_2_O (1:1) mixture. The obtained data are summarized in Table 3. From the results, it is quite evident that concentration of **SM4** on TiO_2_ surface was higher than that of other four dyes. This is in agreement with the experimentally obtained *J*_SC_ values of **SM4**, which is the highest among the three sensitizers.

Interestingly, **SM4** showed the highest values of *V*_OC_ among all dyes, including **N-719**. Due to the site-selective adsorption behavior of the **SM4**, the adsorption of prepared **SM4** may form a better dye coverage to help to passivate the TiO_2_ surface or form an insulating molecular layer composed of prepared dye and thus reduces the recombination due to electron back-transfer between TiO_2_ and ${\mathrm{I}}_{3}^{ -}/{\mathrm{I}}^{-}$. Also, the **SM4** can be adsorbed on the TiO_2_ surface with a higher density than the **N-719** because it has multiple-anchoring groups. This finding clearly shows that the thiazolidine-5-one unit can potentially serve as an efficient electron-accepting platform or metal-free dye in order to increase device performance.

### EIS Studies

**Fig. 10:**
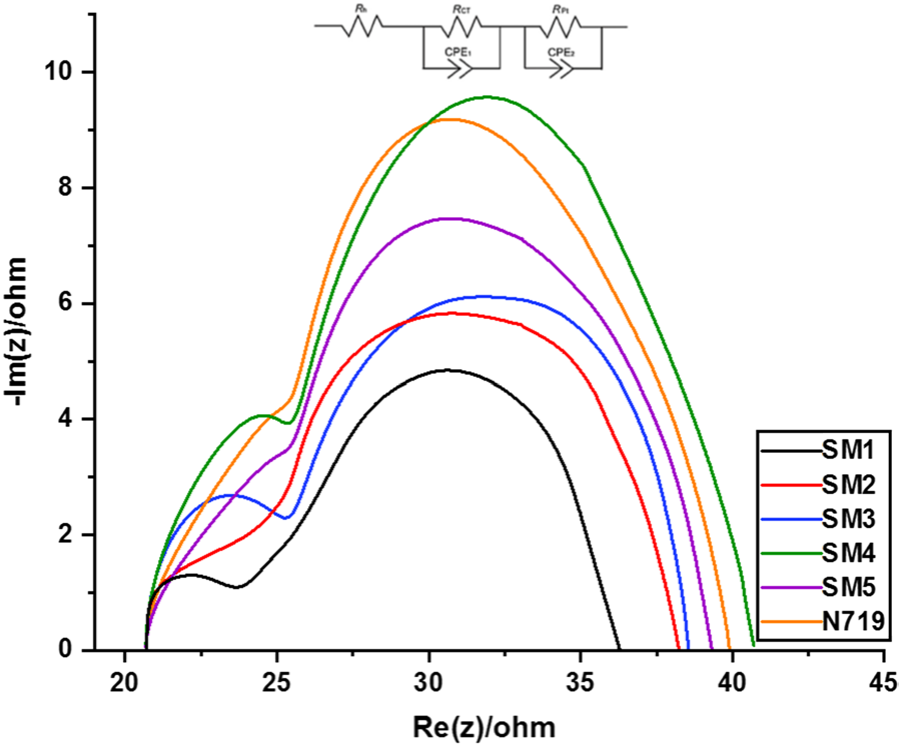
Nyquist plots of SM1-5- and N-719-based devices

Electrochemical impedance spectroscopy (EIS) is a powerful technique used to estimate the charge recombination in DSSCs [57, 58]. Figure 10 shows the Nyquist plots of the fabricated dyes **SM1-5** along with standard **N-719** and their equivalent circuit (inset Fig. 9). The EIS spectrum of a cell exhibited two semicircles in the Nyquist plots. Generally, the first semicircle at high frequencies represents the redox charge transfer resistances at the Pt/electrolyte interface (*R*_pt_). The second semicircle at higher frequencies is related to the charge transfer resistances at the interface between TiO_2_/dye/electrolyte (*R*_CT_). Usually, a larger R_CT_ value indicates an increased charge recombination resistance and therefore a larger open-circuit photovoltage [58, 59].

The charge recombination resistance of these dyes (*R*_CT_) corresponding to the diameter of the middle-frequency semicircle was calculated to decrease in the order **SM4** (20.32 Ω), **N-719** (18.13 Ω), **SM5** (16.05 Ω), **SM3** (15.12 Ω), **SM2** (14.17 Ω) and **SM1** (12.26 Ω), in good agreement with the order photovoltage data. The result indicates that dye **SM4** with thiazolidine-5-one can more effectively reduce charge recombination at the TiO_2_/dye/electrolyte interface than other dyes and **N-719** dye. These results were also consistent with the *V*_OC_ of the DSSCs.

## Conclusion

In conclusion, five new di-anchored metal-free organic dyes **SM1-5** were effectively developed, produced, and characterized. Optoelectronic, electrochemical, and molecular modeling studies show that structures **SM1-5** meet all the requirements for acting as photosensitizers. Additionally, theoretical investigations on compounds **SM1-5** show that the electron density shifts significantly from the triphenylamine donor to the acceptor/anchoring group through the π-spacer. The device fabricated with the **SM4** sensitizer displayed the highest photon to current efficiency (PCE of 6.09%). Its *J*_SC_ and *V*_OC_ were 14.13 mA cm^−2^ and 0.624 V, respectively. The inclusion of a strongly electron-withdrawing dimalononitrile unit on each side of the thiazolidine-5-one core accounts for its improved performance. The findings clearly imply that the thiazolidine-5-one unit connected to the malononitrile core might be an outstanding electron acceptor system for metal-free dyes in order to increase power conversion efficiency. Molecular engineering is being used to develop DSSCs with organic dye-sensitized solar cells depending on the framework of **SM4**, with the goal of improving solar performance.

## Supplementary Information

**Additional file 1.** Supplementary figures.

## Data Availability

All data supporting the conclusions of this article are included within the article and supplementary document.
