# Supplementary material for: Synthesis of Novel Triphenylamine-Based Organic Dyes with Dual Anchors for Efficient Dye-Sensitized Solar Cells
Source: Nanoscale Res Lett. 2022 Aug 4;17:71. doi: 10.1186/s11671-022-03711-6 (PMC9352838; doi:10.1186/s11671-022-03711-6)
Supplement: Supplementary file 1 — Additional file 1. Supplementary figures. [file 11671_2022_3711_MOESM1_ESM.docx]

**Synthesis of novel triphenylamine-based organic dyes with dual anchors for efficient dye-sensitized solar cells.**

Samar E. Mahmoud, Ahmed A. Fadda, Ehab Abdel-Latif, Mohamed R. Elmorsy*

*Department of Chemistry, Faculty of Science, Mansoura University, El-Gomhoria Street, 35516 Mansoura, Egypt.*

*^*^ Corresponding author: E-mail:* [m.r.elmorsy@gmail.com](mailto:m.r.elmorsy@gmail.com)

**1. Spectral analysis:**


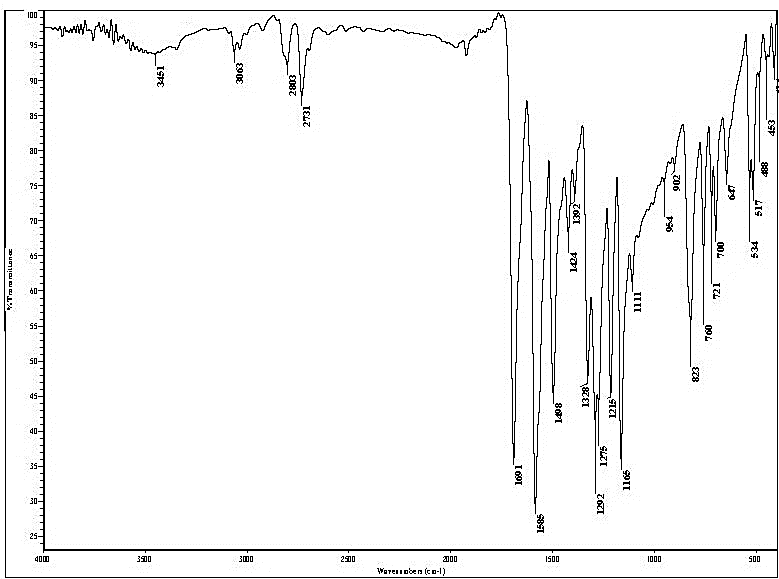

**Figure (1): IR spectrum of compound (3)**


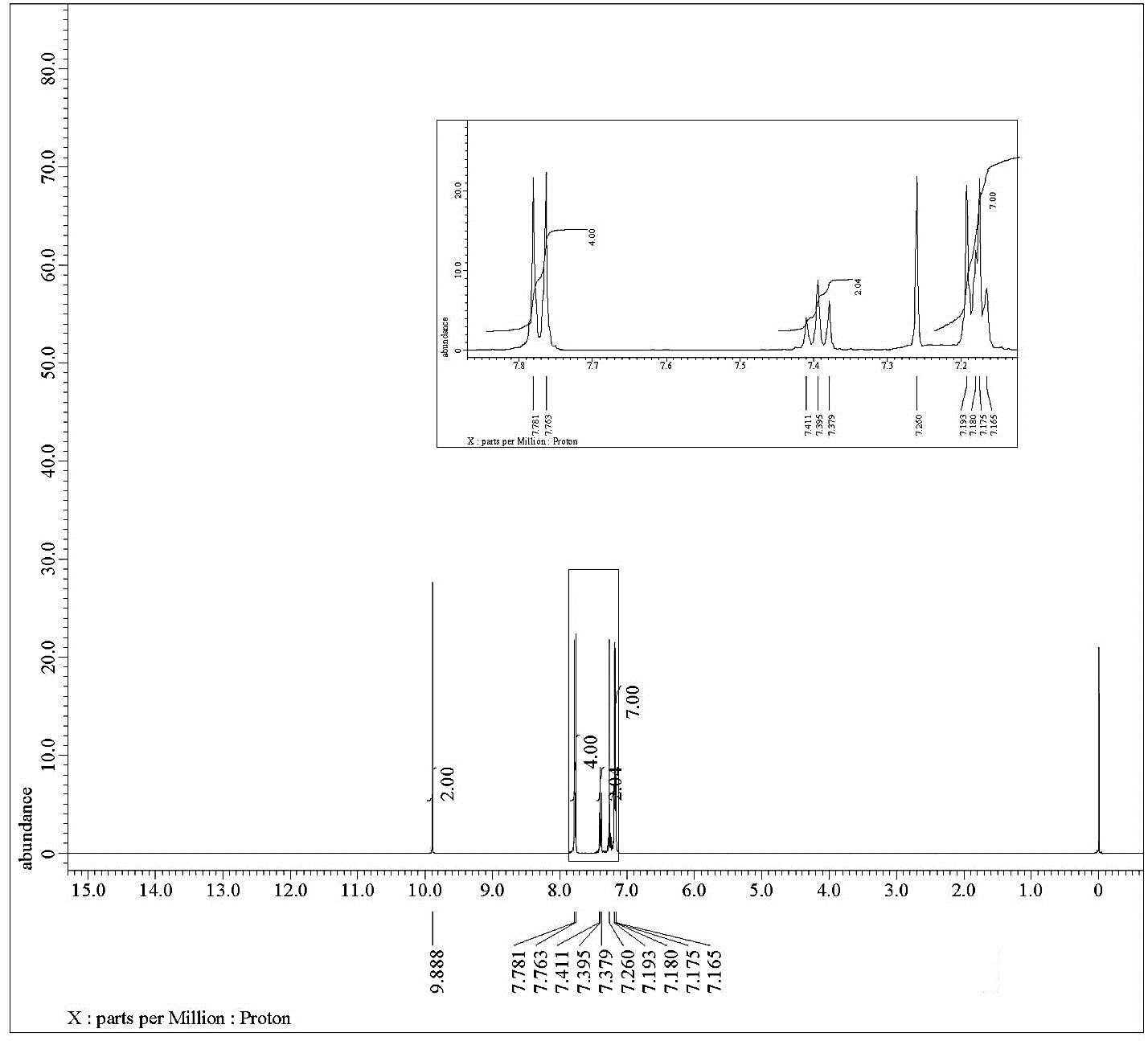

**Figure (2): ^1^HNMR spectrum of compound (3)**


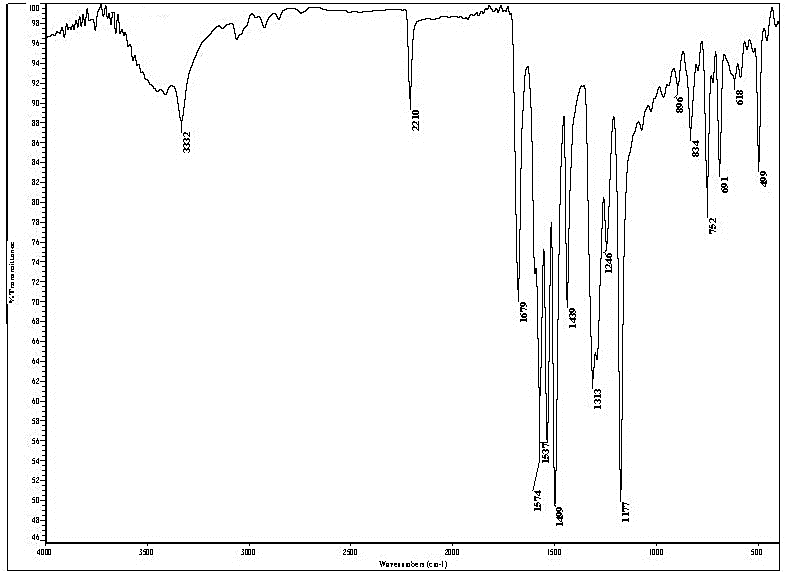

**Figure (3): IR spectrum of sensitizer SM-1**


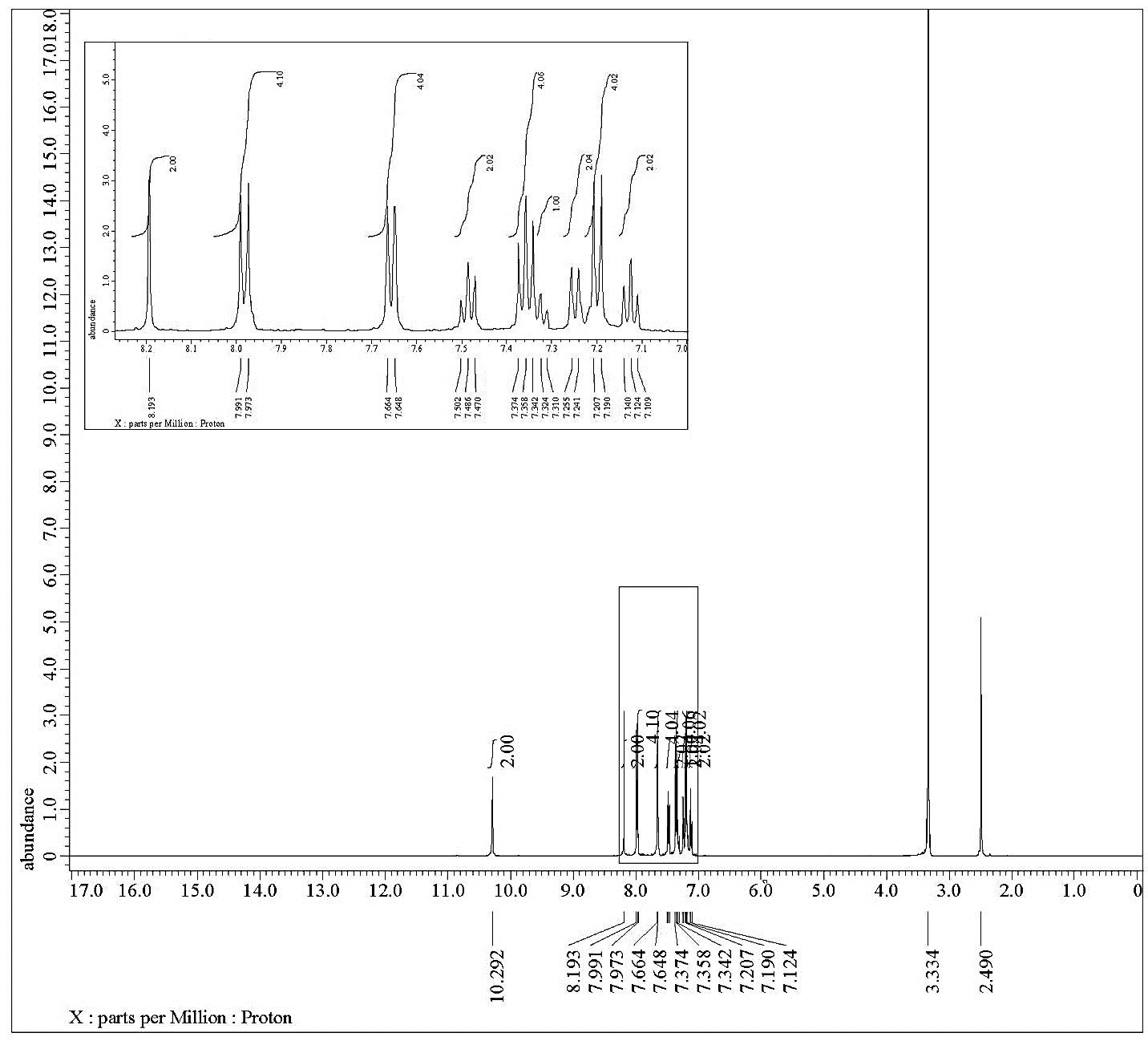

**Figure (4): ^1^HNMR spectrum of sensitizer SM-1**


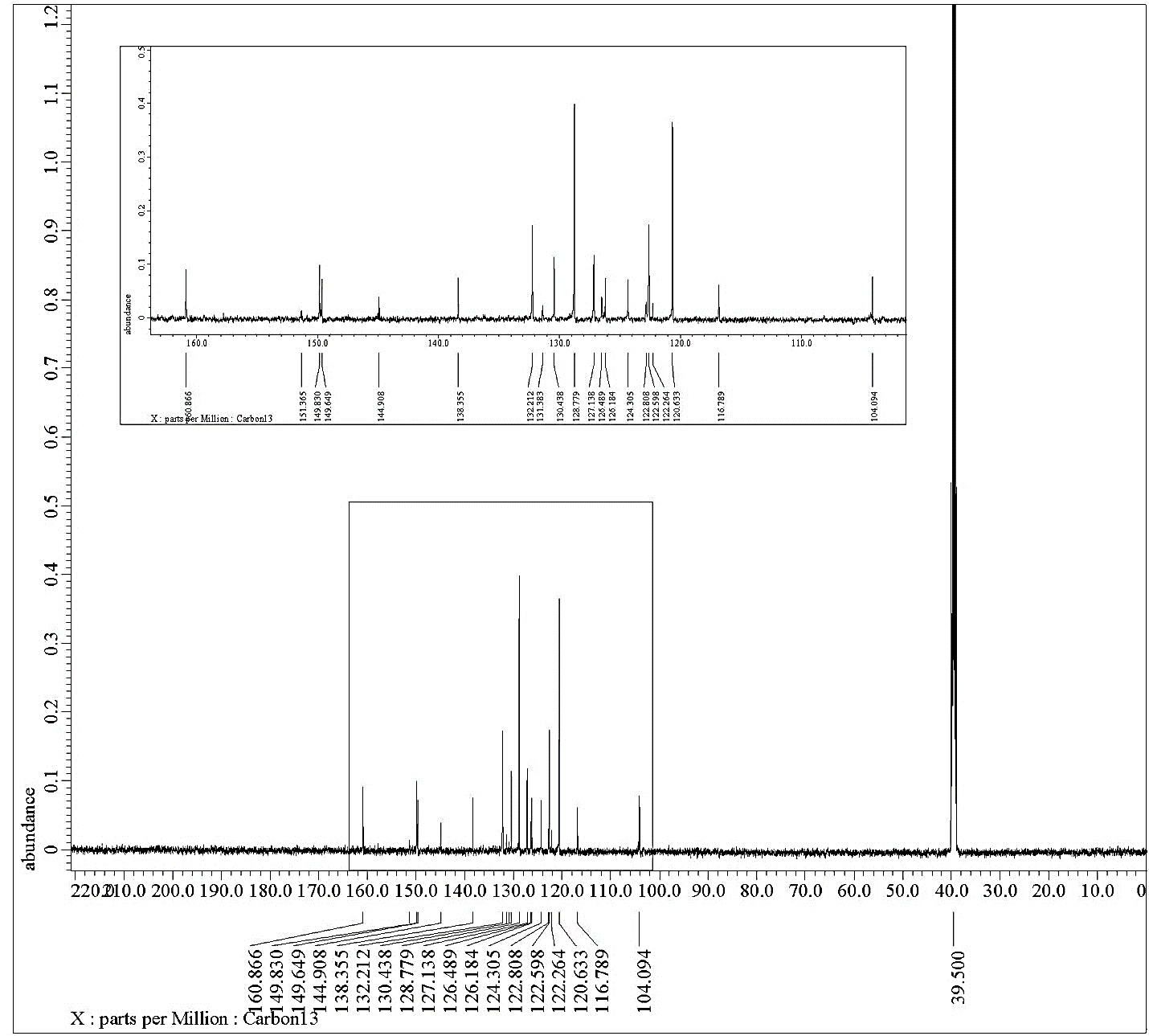

**Figure (5): ^13^C NMR spectrum of sensitizer SM-1**

**Figure (6): Mass spectrum of sensitizer SM-1**


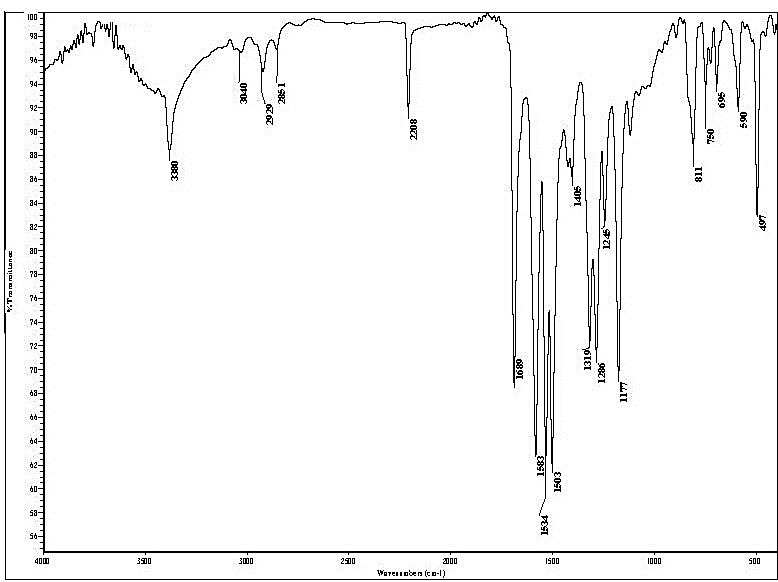

**Figure (7): IR spectrum of sensitizer SM-2**


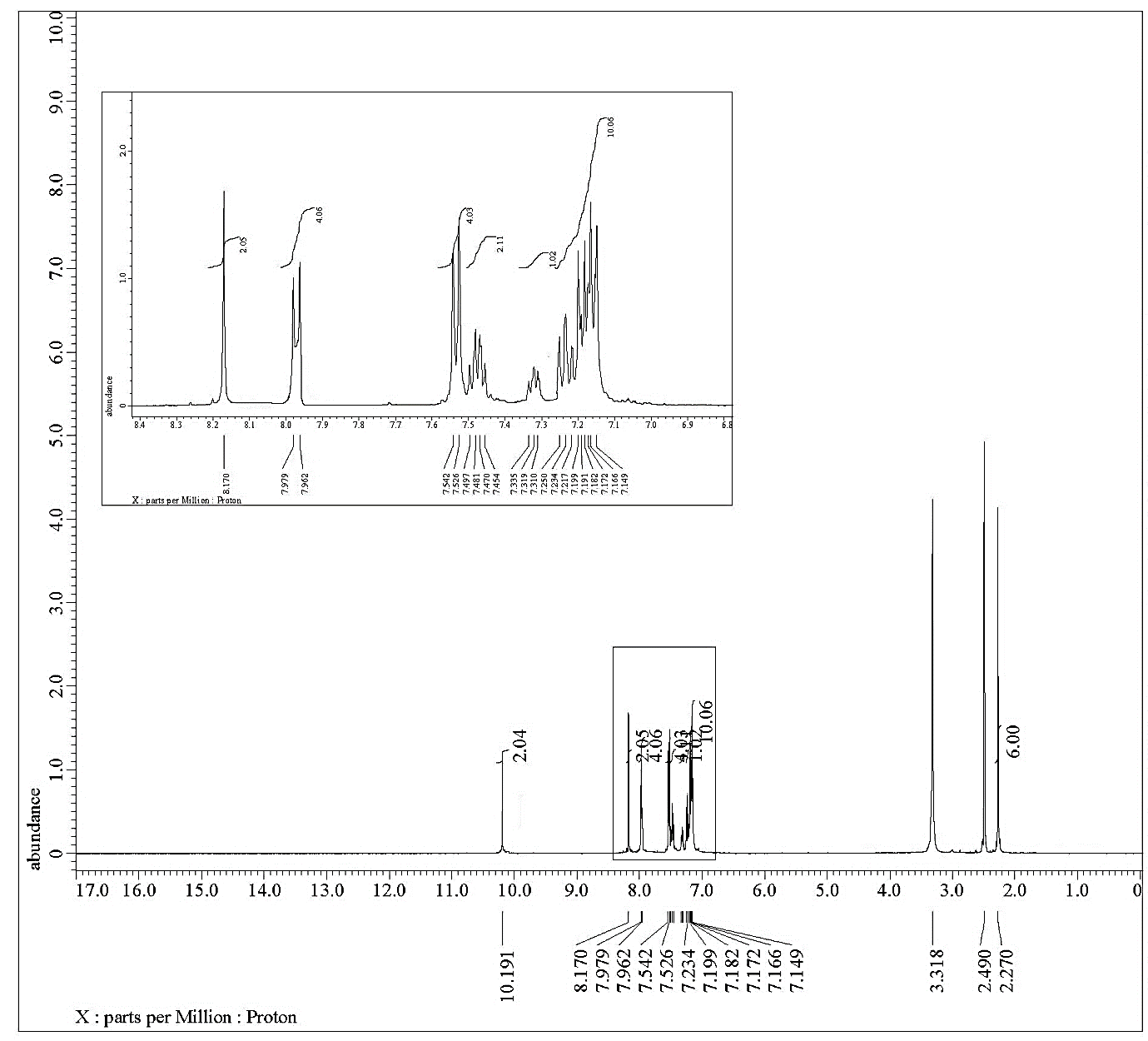

**Figure (8): ^1^HNMR spectrum of sensitizer SM-2**


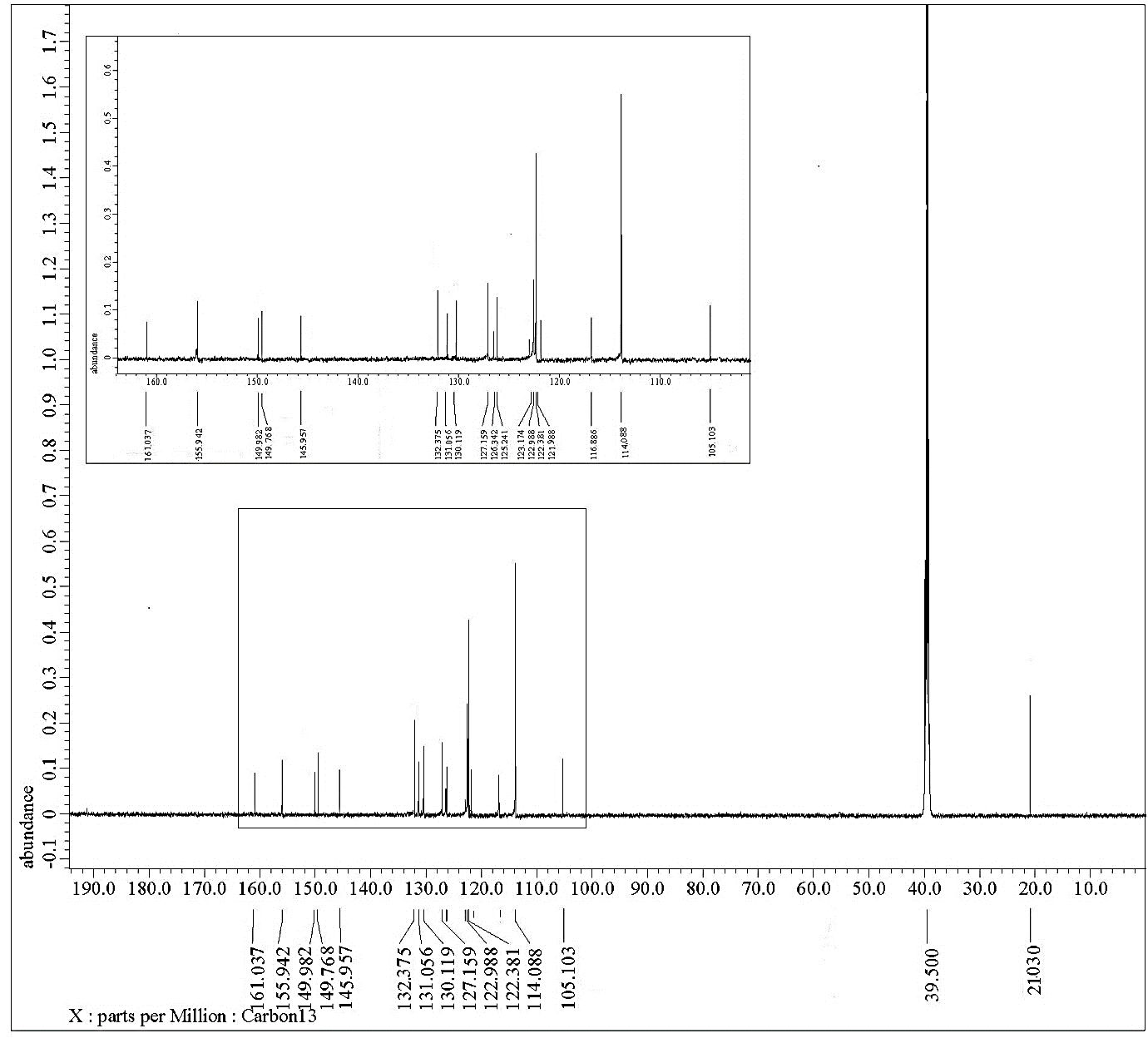

**Figure (9): ^13^C NMR spectrum of sensitizer SM-2**

**Figure (10): Mass spectrum of sensitizer SM-2**


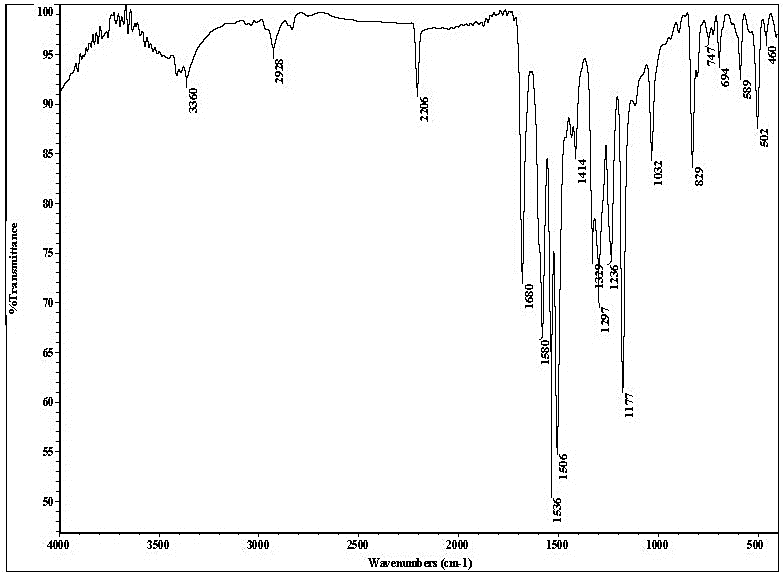

**Figure (11): IR spectrum of sensitizer SM-3**


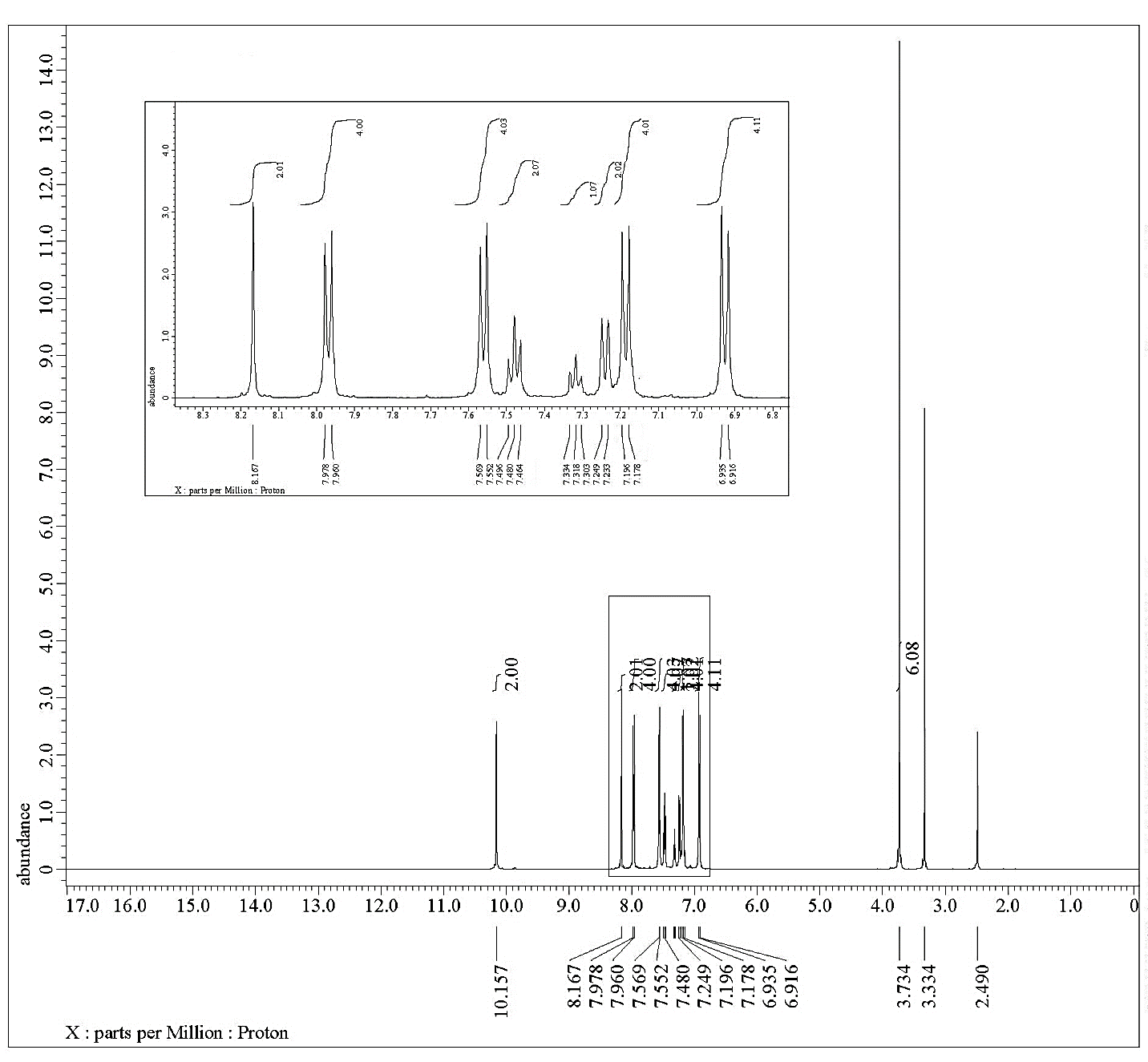

**Figure (12): ^1^HNMR spectrum of sensitizer SM-3**


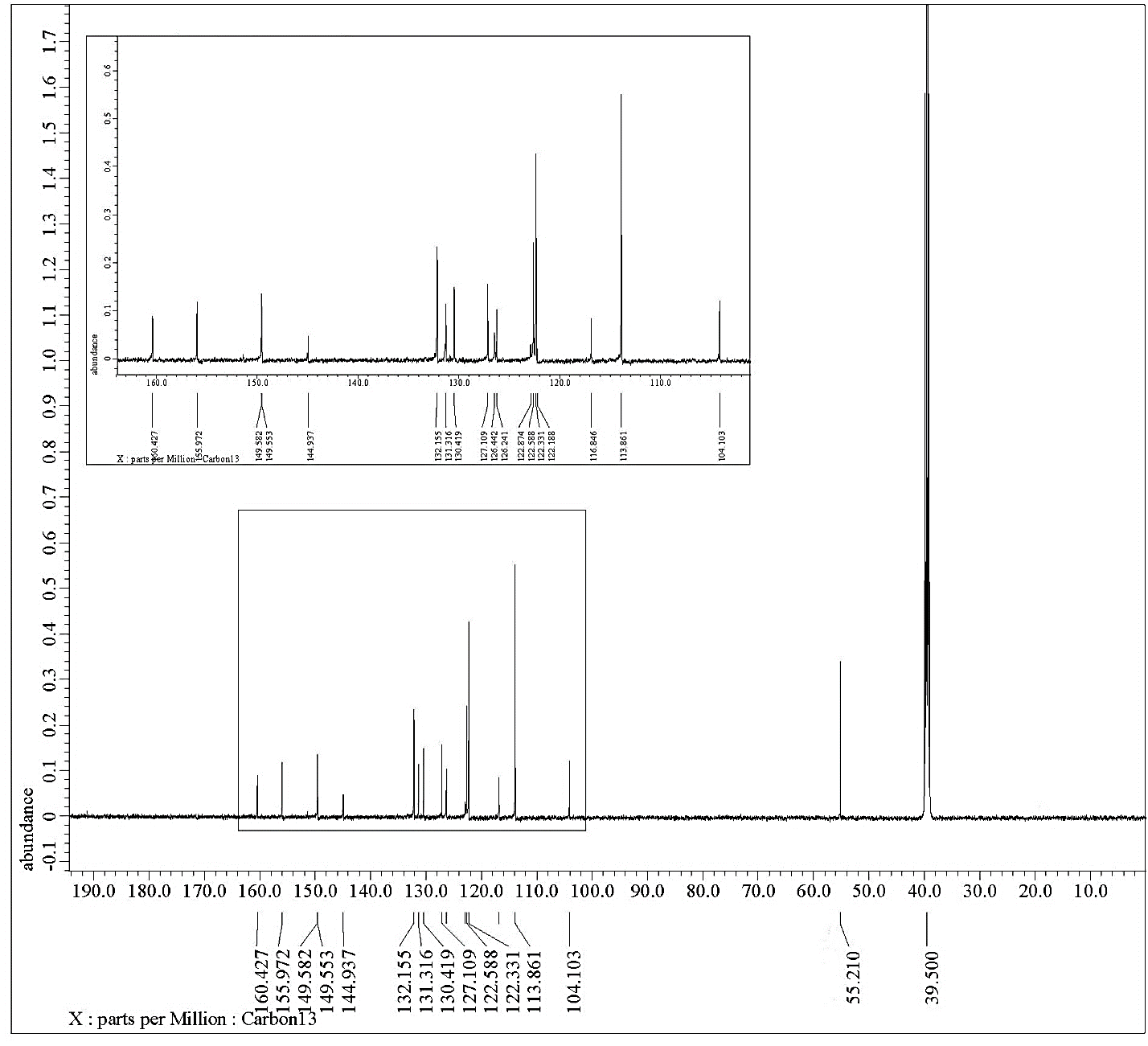

**Figure (13): ^13^C NMR spectrum of sensitizer SM-3**

**Figure (14): Mass spectrum of sensitizer SM-3**


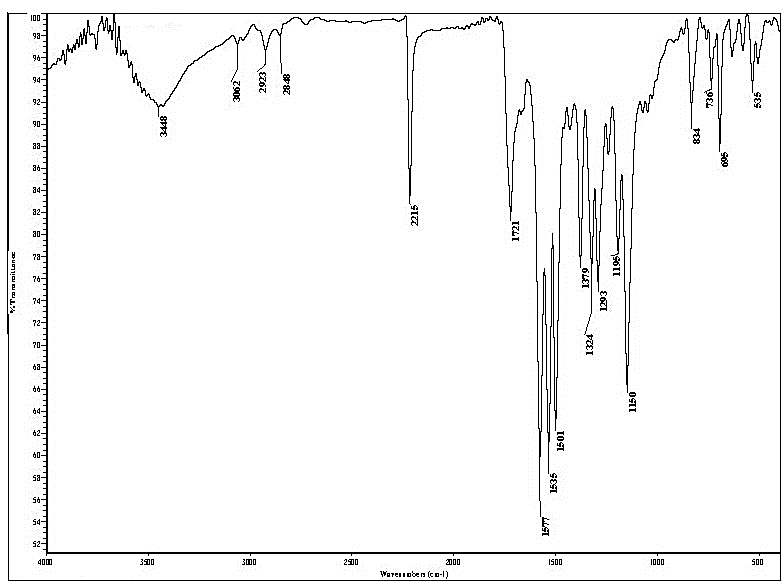

**Figure (15): IR spectrum of sensitizer SM-4**


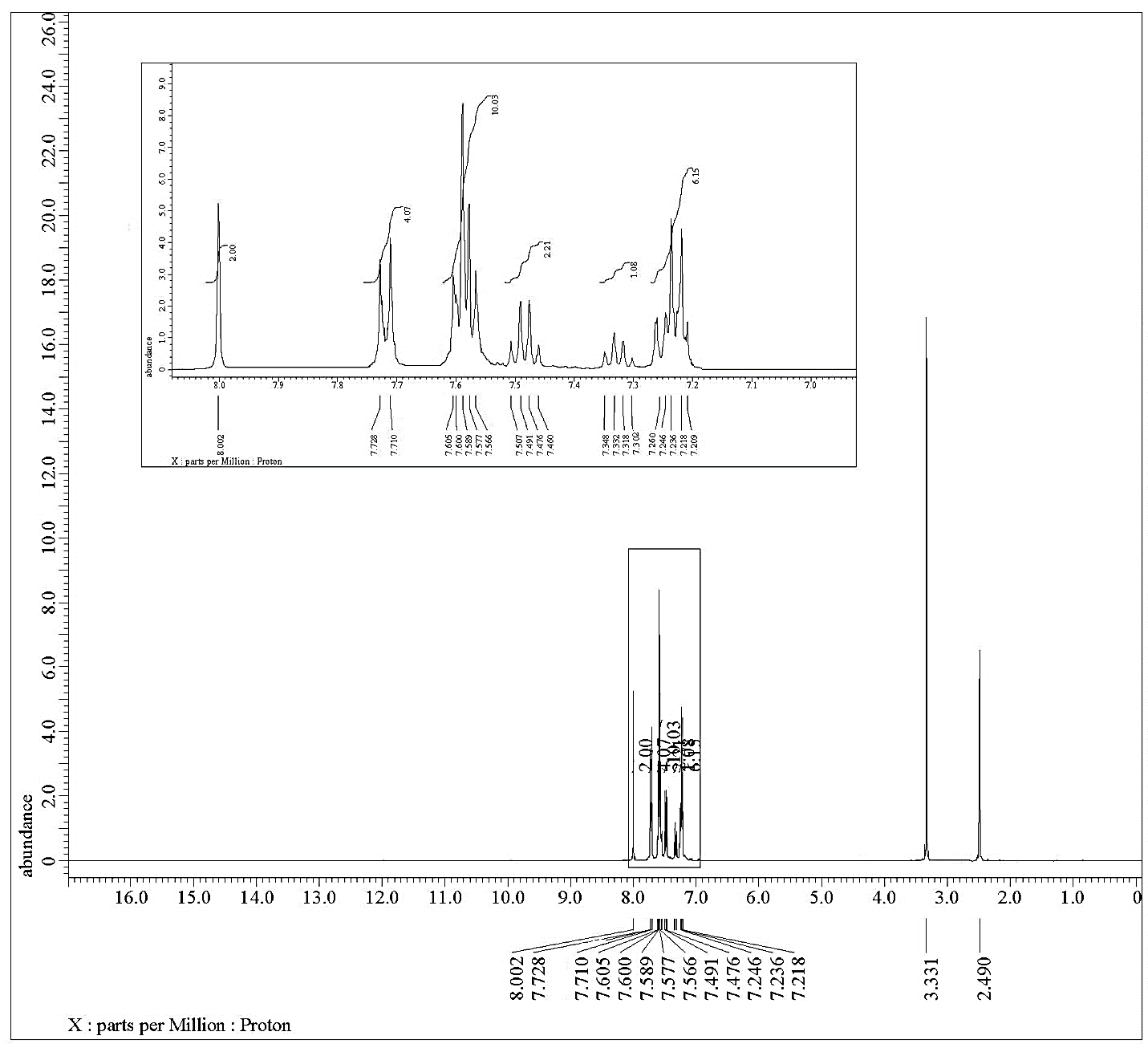

**Figure (16): ^1^HNMR spectrum of sensitizer SM-4**


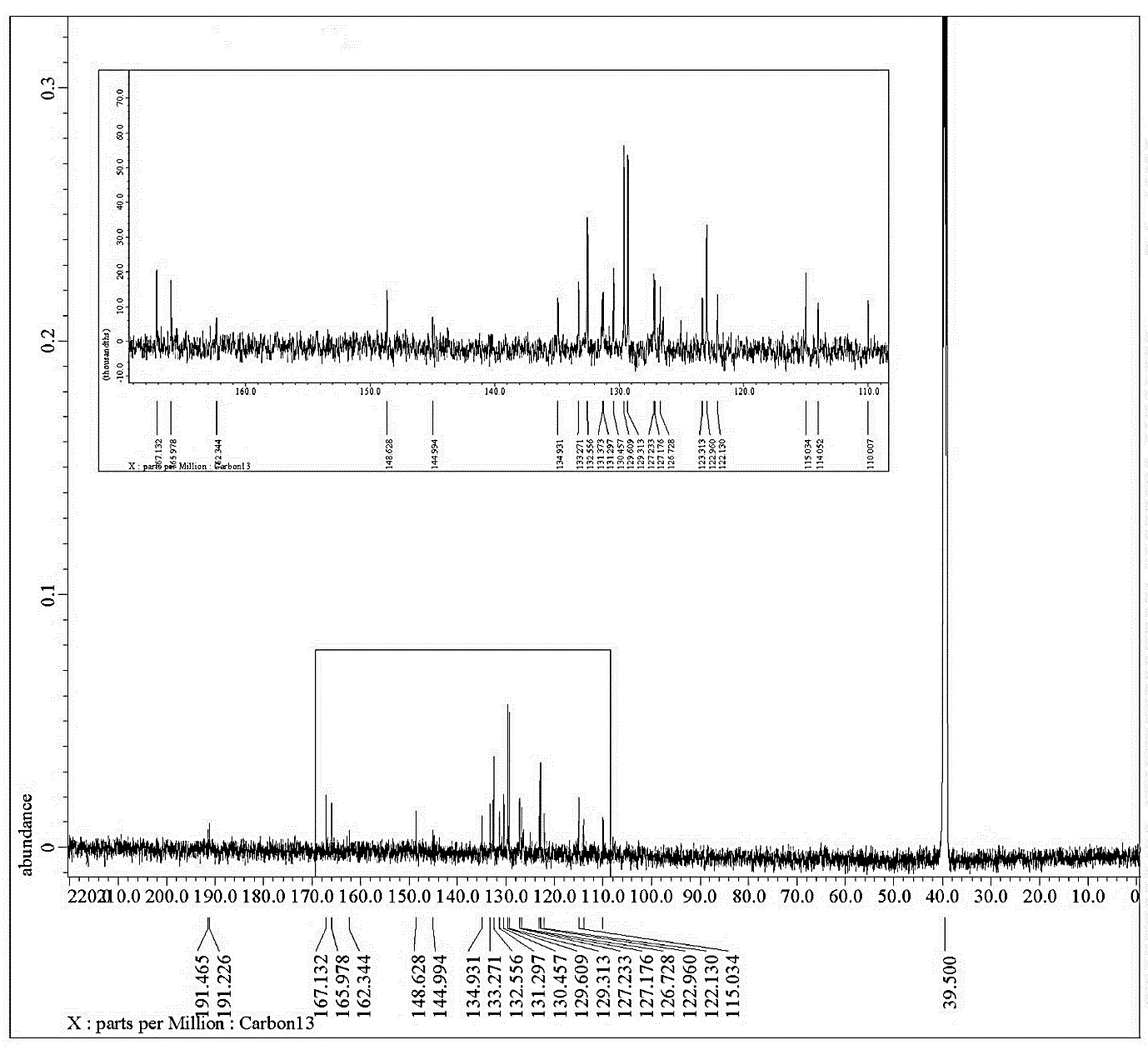

**Figure (17): ^13^C NMR spectrum of sensitizer SM-4**

**Figure (18): Mass spectrum of sensitizer SM-4**


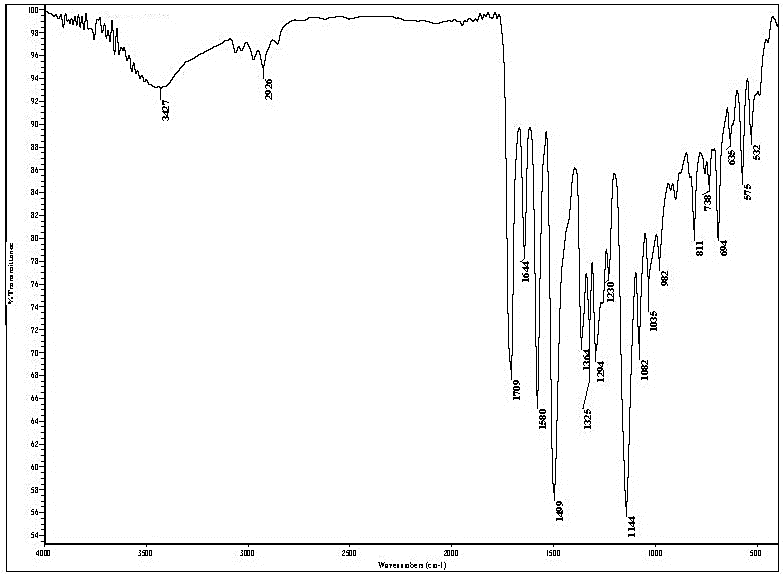

**Figure (19): IR spectrum of sensitizer SM-5**


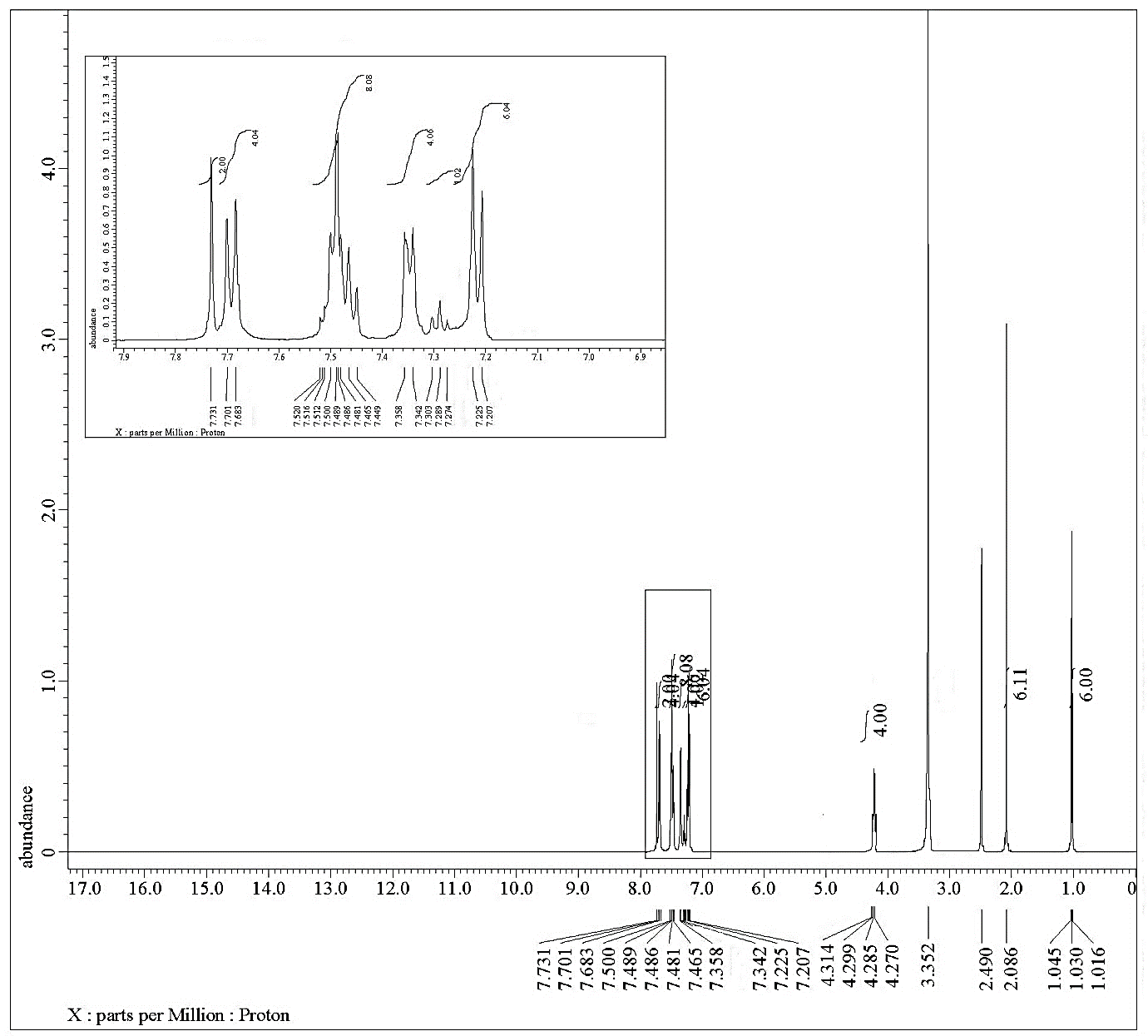

**Figure (20): ^1^HNMR spectrum of sensitizer SM-5**

**
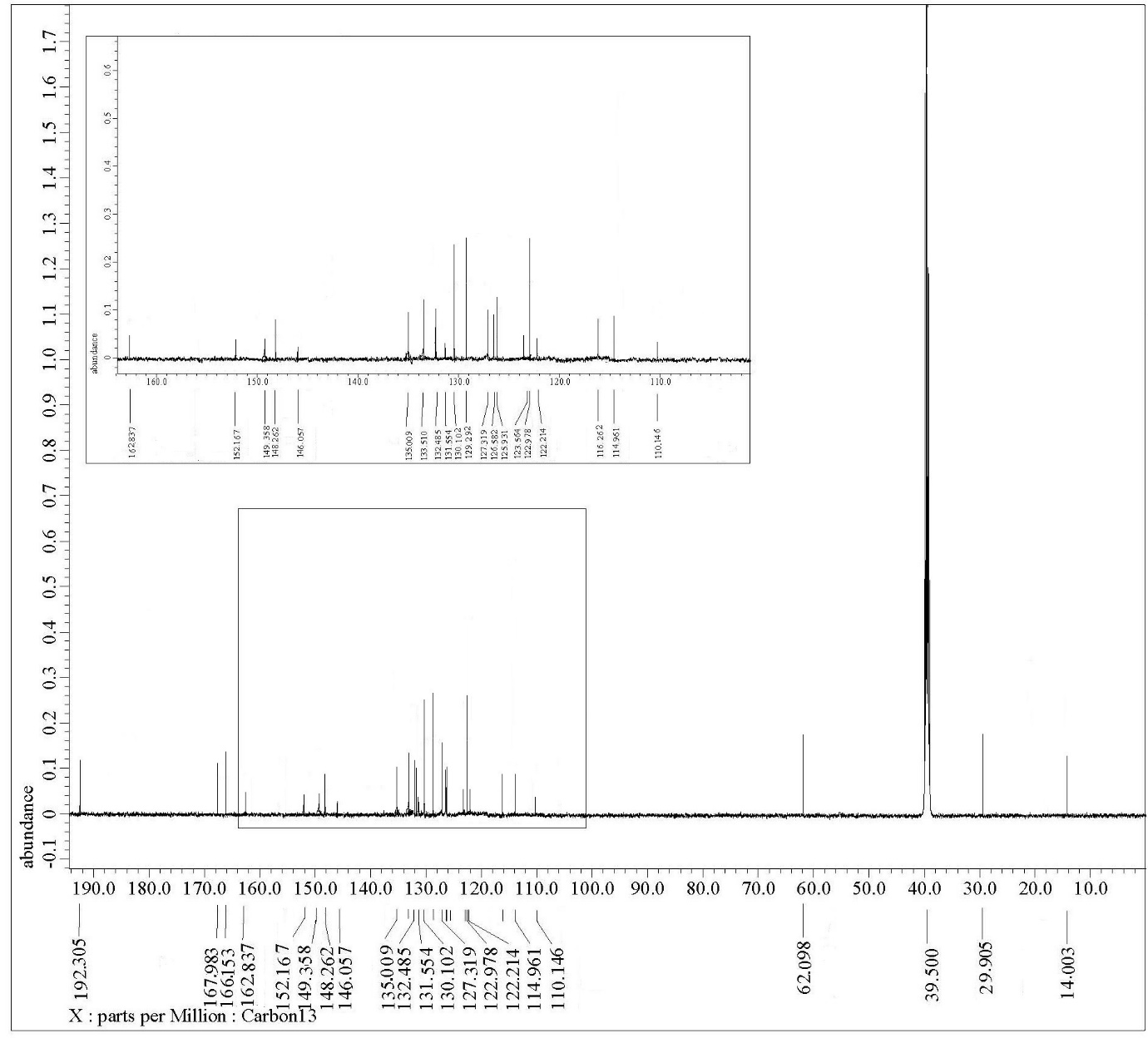
**

**Figure (21): ^13^C NMR spectrum of sensitizer SM-5**

**Figure (22): Mass spectrum of sensitizer SM-5**

**2. CV studies**

The CV measurements were performed by using the three-electrode system, consisting of dye casted on glassy carbon electrode as the working electrode, Pt electrode as a counter and Ag/AgCl as a reference electrode. All three electrodes were immersed in the acetonitrile solution consisting of 0.1 M tetrabutyl ammonium hexafluorophosphate as supporting electrolyte and data were recorded at a scan rate of 100 mV/s. The obtained CV curves of **SM1-5** are presented in **Fig 23.**


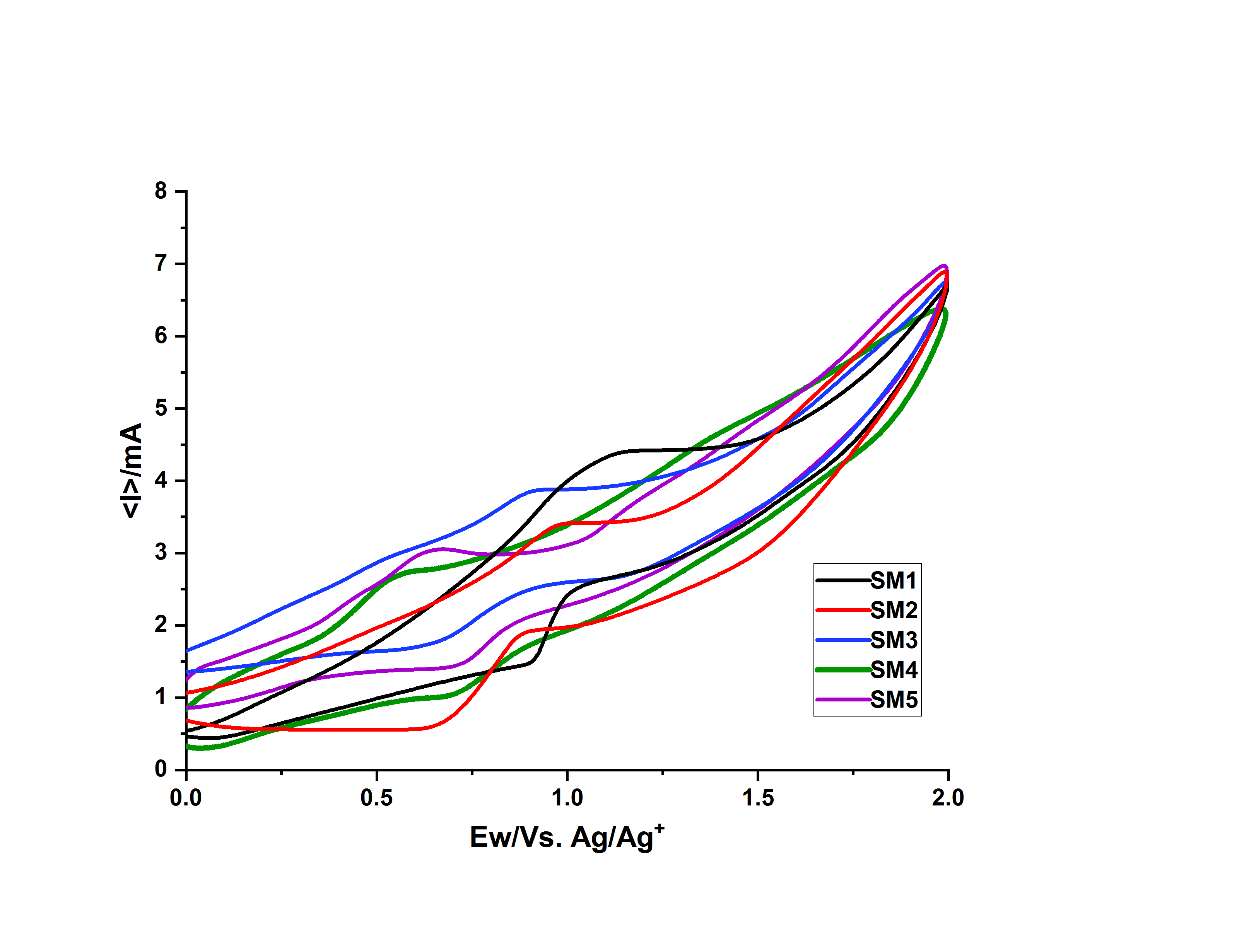


**Figure (23):** CV curves of **SM1-5**

**3. Fabrication of dye-sensitized solar cell**

Photovoltaic measurements were made on sandwich cells, which were prepared using TiO_2_ coated working electrodes and platinum coated counter electrodes and were sealed using a 40 μm Syrlyn spacer through heating of the polymer frame. The redox electrolyte (Solaronix, Iodolyte HI-30) consisted of a solution of 0.6 M DMPII, 0.05 M I_2_, 0.1 M LiI and 0.5 M TBP in acetonitrile.

**4. Photovoltaic measurements**

Photovoltaic measurements of sealed cells were made by illuminating the cell through the conducting glass from the anode side with a solar simulator at AM 1.5 illuminations (light intensity: 100 mW cm^−2^).

**5. Electrochemical impedance spectroscopy (EIS)**

The electrochemical impedance spectra were measured with an impedance analyzer potentiostat (Bio-Logic) under illumination using a solar simulator. The electrical impedance spectra were fitted using Z-Fit software (Bio-Logic).

**6. Molecular Modeling**

Equilibrium molecular geometries of **SM1-5** calculated using the Becke's three parameter hybrid functional, Lee-Yang-Parr's gradient corrected correlation functional (B3LYP) and (6-311G (d, p)) [1, 2, 3, 4]. The geometry optimization calculations were followed by energy calculations using time-dependent density functional theory (TD-DFT) utilizing the energy, functional B3LYP and the basis set 6-311G (d, p), implemented in Gaussian 09.

**References**

[1] G. Melikian, F. Rouessac, C. Alexandre, A convenient synthesis of substituted 3-pyrrolin-2-ones from α-cetols, Synth. Commun. 23 (1993) 2631-2638, https://doi.org/10.1080/00397919308013792.

[2] A. D. Becke, Density-functional exchange-energy approximation with correct asymptotic behavior, Phys. Rev. A 38 (1988) 3098, https://doi.org/10.1103/PhysRevA.38.3098.

[3] C. T. Lee, W.T. Yang, R.G. Parr, Phys. Rev. B. **1988**, 37, 785

[4] N. Godbout, D. R. Salahub, J. Andzelm, E. Wimmer, Optimization of Gaussian-type basis sets for local spin density functional calculations. part 1. Boron through neon, optimization technique and validation. Can. J. Chem. 70 (1992) 560-571, https://doi.org/10.1139/v92-079@cjc-uc-0101.
